# Phenolic and Antioxidant Characterization of Fruit By-Products for Their Nutraceuticals and Dietary Supplements Valorization under a Circular Bio-Economy Approach

**DOI:** 10.3390/antiox13050604

**Published:** 2024-05-14

**Authors:** Cristina Terenzi, Gabriela Bermudez, Francesca Medri, Lara Davani, Vincenzo Tumiatti, Vincenza Andrisano, Serena Montanari, Angela De Simone

**Affiliations:** 1 Department for Life Quality Studies, University of Bologna, Corso D’Augusto 237, 47921 Rimini, Italy; cristina.terenzi2@unibo.it (C.T.); gabriela.bermudez2@unibo.it (G.B.); francesca.medri2@studio.unibo.it (F.M.); lara.davani2@unibo.it (L.D.); vincenzo.tumiatti@unibo.it (V.T.); serena.montanari5@unibo.it (S.M.); 2 Department of Drug Science and Technology, University of Torino, Via P. Giuria 9, 10125 Torino, Italy

**Keywords:** agri-food by-products, polyphenols, antioxidant activity, circular economy, waste valorization, sustainable ingredients

## Abstract

Agri-food by-products, obtained as waste from the food industry, negatively impact the global economy and the environment. In order to valorize waste materials from fruit juices and tomato sauces as upcycled materials rich in health-promoting compounds, they were characterized in terms of polyphenolic and protein content. The results obtained were compared with those collected for their final products. The recovery of polyphenols was performed via ultrasound-assisted extraction (UAE). A high-performance liquid chromatography–diode array detector (HPLC-DAD) method was developed and validated to depict the quali-quantitative polyphenolic profile of both the by-products and the final products. The antioxidant capacity of the resulting extracts was characterized by UV-Vis spectrophotometric assays in terms of total phenolic content (TPC) and total antioxidant status (TAS). Moreover, the protein content was assessed with the Kjeldahl method too. The results highlighted a significant quantity of polyphenols remaining in peach, apricot, and apple by-products, which were able to exert an antioxidant activity (in the range of 4.95 ± 5.69 × 10^−1^ to 7.06 ± 7.96 × 10^−1^ mmol Trolox 100 g^−1^ of dry weight (DW) sample). Conversely, the tomato by-products were highly rich in proteins (11.0 ± 2.00 to 14.4 ± 2.60 g of proteins 100 g^−1^ DW). The results proved that all by-products may potentially be sustainable ingredients with nutritional and functional value in a circular bio-economy prospect.

## 1. Introduction

The increasing waste generation and the limited availability of natural resources have prompted the scientific community to investigate the possibilities of recovering valuable compounds from different waste streams [1]. Food processing, one of the most important industrial sectors in the world, produces large and accumulative quantities of waste, negatively impacting the global economy and the environment [2]. To overcome this problem, a circular economy approach has been proposed as a regenerative growth model that could give back to the planet more than it takes [3] by reusing and recycling existing materials for as long as possible [4]. Agri-food by-products, comprising pomaces, peels, stalks, seeds, seed hulls, and coats obtained from specific food-processing techniques, such as pulping, peeling, straining, and branching [5], have been studied as prospective circular materials due to their high valorization potential as sources of valuable biomolecules [6].

In this work, the attention has been focused on by-products obtained from fruit juice production. The different parts that are separated or transformed during several processing steps lead to by-products with a distinct composition and distribution of biomolecules [1]. As regards fruit by-products involved in this study, we considered pomaces obtained from pulping: apple pomace is a by-product that consists of pulp, peels, seeds, and stalks generated from apple juice production [7] and represents around 25% of the processed apple [1]; peach and apricot pomaces consist only of the pulp and the peel tissues and account for approximately 10% of the initial fruit weight used for juice production [8,9]. Moreover, tomato by-products from tomato paste production, consisting primarily of seeds (60%) and skin (40%) [10], which account for almost 5% of waste [11], were also characterized.

All of these by-products are potential sources of value-added compounds that can be recovered from the fruit waste, including (i) polyphenols [7,8,9,12], which are of the most importance due to a wide bioactivity spectrum positively supporting human health [6], acting primarily as antioxidants and photo protectors able to fight oxidative stress involved in aging and degenerative diseases [13,14,15,16], (ii) plant-based proteins of nutritional value [10,17], or as source of bioactive peptides [18,19].

In light of this, the present study was focused on the chemical and functional characterization of different waste materials from fruit processing, which were supplied by the biggest Romagna agri-food consortium and an industrial cooperative company specialized in the design, supply, and development of private label products. Therefore, this study, by collating the attention of a huge agriculture product market, was oriented towards the characterization, both in terms of polyphenols and protein content, of the chemical profile of fruit (peaches, apricots, apples, and tomatoes) by-products and final products generated from the industrial processing of fruit juices and tomato pastes. An extractive method based on ultrasound-assisted extraction (UAE) was developed to obtain extracts rich in polyphenols. A high-performance liquid chromatography–diode array detector (HPLC-DAD method was optimized for the characterization of the extracts in terms of polyphenolic content. The separation of 22 phenolic reference standards was indeed validated in terms of sensitivity, linearity, precision, specificity, and accuracy (recovery) to characterize the polyphenol content profile of the extracts from a qualitative and quantitative point of view.

The extracts were also characterized by two different UV-Vis spectrophotometric assays: the total phenolic content (TPC), determined by the Folin–Ciocalteu method, and the total antioxidant status (TAS), evaluated by the Trolox equivalent antioxidant capacity (TEAC) assay, both performed to assess the antioxidant activity. On the other hand, the protein content of each sample was assessed following the Kjeldahl method.

In light of the interesting results obtained, the potential valorization of these waste materials, which are normally discarded, as valuable ingredients for nutraceutical applications was discussed. The depicted unique polyphenolic profile, protein content, and antioxidant properties compared to their respective final product proved that fruit by-products could be potential alternative sources of sustainable bioactive compounds.

## 2. Materials and Methods

### 2.1. Chemicals and Reagents

Methanol HPLC grade ≥ 99.9%, methanol ≥ 99.8%, acetone HPLC grade ≥ 99.8%, eth-anol and acetic acid ≥ 99.8%, potassium persulfate ≥ 99.0%, ABTS (2,2-azinobis-(3-ethylbenzothiazoline-6-sulfonate), Trolox^®^ (6-hydroxy-2,5,7,8-tetramethyl-3,4-dihydrochromene-2-carboxylic acid), sodium hydroxide ≥ 98% in pellets, sodium carbonate ≥ 99.5%, gallic acid ≥ 95%, protocatechuic acid ≥ 97%, trans-cinnamic acid ≥ 98.0%, caffeic acid ≥ 98.0%, p-coumaric acid ≥ 98.0%, ferulic acid ≥ 99.5%, (+)-catechin ≥ 99.0%, isoquercitrin ≥ 98.0%, daidzein ≥ 97.0%, genistein ≥ 97.0%, and phloridzin dihydrate ≥ 98.5% were purchased from Sigma-Aldrich (Taufkir-chen, Germany); chlorogenic acid ≥98.0% was purchased from Apollo Scientific (Bredbury, UK); and Quer-citrin ≥ 98% was purchased from Cayman Chemical (Ann Arbor, MI, USA). Folin–Ciocalteu reagent was purchased from VWR Chemicals (Darmstadt, Germany); copper (II) sulfate pentahydrate and potassium sodium tartrate tetrahydrate EMSURE^®^ were purchased from Merck (Darmstadt, Germany); (−)-epicatechin ≥ 97.0%, hesperetin ≥ 97.0%, and apigenin ≥ 97.0% were purchased from TCI (Zwijndrecht, Belgium); hyperoside ≥ 92.0% was purchased from HWI group (Rulzheim, Germany); (+)-rutin trihydrate ≥ 97% was purchased from Alfa Aesar (Haverhill, MA, USA); myricetin ≥ 98%, naringenin ≥ 97% and kaempferol ≥ 98.0% were purchased from ThermoFisher (Kandel, Germany); and naringenin chalcone ≥ 95% was purchased from PhytoLab (Vestenbergsgreuth, Germany). Ultrapure (type 1) water for HPLC was obtained using a Direct-Q^®^ 5 UV system (Merck, Darmstadt, Germany). VWR Syringe Filter, nylon, 0.45 µm, was purchased from VWR International Srl (Milan, Italy). VWR Syringe Filter, PTFE, 0.22 μm, was purchased from VWR International Srl (Milan, Italy).

### 2.2. Equipment

The analytical HPLC system employed was Agilent 1260 Infinity, equipped with ChemStation software 3D system Rev. B 04.03, G1312C 1260 Bin Pump VL, and G1315D 1260 DAD VL detector (Agilent, Santa Clara, CA, USA), a Kinetex XB-C18 column (4.6 mm × 150 mm, 5 μm, 100 Å) (Phenomenex, Torrance, CA, USA). The Spectrophotometer: Jasco UV-VIS V-630 (Jasco Europe, Lecco, Italy). Ultrasound Bath: Elmasonic S 40 H (GEASS S.R.L., Turin, Italy). The centrifuge: Awel International MF 20-R multifunction centrifuge (MedWrench, East Point, GA, USA). Freeze-dryer: Alpha 1-4 LO plus (Martin Christ, Harz, Germany). Rotatory evaporator: IKA Rotary Evaporators RV 10 basic (IKA-Werke GmbH & Co. KG, Staufen im Breisgau, Germany).

### 2.3. Samples

Materials derived from apricot, peach, and apple juice and tomato sauce processing lines were supplied by Fruttagel S.C.p.A (Alfonsine, Italy). Samples were freeze-dried by the supplier (Figures S1–S4) and subjected to grinding in 2 steps. During the first one, a domestic mixer was used to break the solid materials and reduce particle size. The following consisted of a second milling (IKA Tube Mill 100 control) to obtain fine powders, which were subsequently cryo-lyophilized for 24 h (−60 °C) and stored at −20 °C until use.

Fruit by-products (ByP) and final products (FinalP) under study are listed in Table 1.

The products were defined as biological or conventional according to the following descriptions. The conventional products can be obtained using active compounds as reported in the Reg. (CE) n. 396/2005 [20]. On the other hand, the biological products are obtained by cultivation practices regulated by Reg. (CE) 848-2018 [21]. This latter has the final aim to minimize the chemical treatments by also applying microorganisms or insects able to combat in turn some harmful ones. In this case the chemicals used are selected in agreement with their environmental impact.

### 2.4. UAE of Polyphenols from Agri-Food Samples

About 0.5 g of each lyophilized sample were weighed (n = 3), to which 4 mL of a mixture 50:50 methanol:water (containing 3.4% acetic acid) (Solution A) was added. The suspension was vortexed for 1 min and subjected to an ultrasonic bath (Elmasonic S 40 H) at 25 °C for 15 min. The solution was centrifuged at 3680× *g* (4400 rpm) at 20 °C for 15 min, and the supernatant collected in a 50 mL falcon tube. The procedure was repeated three times. The residual biomass was subjected to a second extraction step with 4 mL of a 70:30 acetone:water solution (Solution B), vortexed for 1 min, and subjected to UAE at 25 °C for 15 min. Then, the solution was centrifuged at 3680× *g* (4400 rpm) at 20 °C for 15 min and the supernatant placed in a 50 mL falcon tube. This extractive procedure was performed twice more. Once all 6 supernatants were collected, the last centrifugation at 3680× *g* (4400 rpm) at 20 °C for 5 min was performed, and the residual particles were removed. The obtained extracts were filtered through a nylon 0.45 μm filter, placed in a round bottom flask, and dried with a rotatory evaporator at 40 °C and with cryo-lyophilization for 24 h. Dried extracts were weighed for gravimetric determination and then stored in plastic Eppendorfs at −80 °C until use.

### 2.5. Gravimetric Determination (Yield of Extraction)

Gravimetric determination was conducted on all dried extracts obtained with UAE to calculate the yield of extraction (%), as follows:

yield% = (grams of dried extract/grams of sample) × 100

Finally, to perform TPC assay, TAS assay, and HPLC-DAD analysis, the dried extracts were reconstituted with 25 mL of a mixture 50:50 Solution A:Solution B (*v*/*v*), described in Section 2.4, to obtain the sample solutions to test. All the obtained results are reported in Table S1.

### 2.6. Liquid Chromatography–Diode Array Detection (HPLC-DAD) Analysis of Polyphenols

Tables S2 and S3 report the spectroscopic and chromatographic features as well as the concentration of the 22 standard polyphenols whose separation was achieved with HPLC analysis in gradient mode. The column was kept at 20.0 °C ± 0.8. The mobile phase consisted of 100% methanol (solvent A) and 2% acetic acid water solution (solvent B). The optimized gradient was 2–30% A (0–10 min) and 30–46% A (10–65 min) at a flow rate of 1.0 mL min^−1^. The injection volume was 20 μL. Detection was performed at five different wavelengths (λ = 280 nm; λ = 320 nm; λ = 370 nm; λ = 360 nm; λ = 250 nm), which accounted for all the maximum absorptions of different standards in the mixture. Chromatographic peaks were identified by comparison of elution order, retention times (RT), and UV-Vis absorption spectra with those of standards. Compounds for which no standards were available were identified by spectral parameters of the phenolic class/family and quantified using the calibration curves of a member of the phenolic class with similar spectra, as described by Mesquita and Monteiro [22]. Concerning sample preparation, all extracts (25 mL) were concentrated 10 times and filtered with a PTFE 0.22 μm filter before injection. Then, 20 μL of these solutions were injected into the HPLC-DAD system under the established chromatographic conditions. Fruit samples were extracted in triplicate, and the obtained solutions were analyzed twice daily for polyphenol determination under the chromatographic conditions described. The amount of each polyphenol in each sample was determined by interpolating their peak area into the respective calibration curve. The determination of polyphenols was carried out and expressed in mg 100 g^−1^ of DW of lyophilized sample powder.

#### HPLC-DAD Method Validation

*Sensitivity*. The limit of detection (LoD) and limit of quantification (LoQ) were obtained by considering the standard deviation of signals (LoD = 3.3 × (SD of intercept/m); LoQ = 10 × (SD of intercept/m); SD of intercept = SE of intercept × √N.

*Linearity.* Stock solutions (SS) of each reference standard were prepared in 100% methanol to a concentration of 1.5 mg mL^−1^. Only apigenin was dissolved in 100% ethanol at the same concentration. Several dilutions of each standard were prepared using methanol: water 50:50 with 2% acetic acid (employed to avoid peak tailing). The standard calibration curves were obtained by analyzing each standard in the linearity ranges described in Table S4. Calibration curves were then obtained via linear least-squares regression analysis (R^2^) by plotting the peak areas versus polyphenol concentration.

*Precision*. The intra-day precision was evaluated by analyzing the mean concentration of the linearity range of each compound and diluted in 50:50 MeOH:H_2_O 2% acetic acid on the same day (n = 3) (Table S6). The inter-day precision was obtained by analyzing the same solutions two times a day on three different days for one week (n = 6) (Table S5).

*Accuracy (Recovery)*. The % recovery was carried out by adding a 22 polyphenol standard mixture at a fixed concentration (reported in Table S6) in a vegetal matrix before and after being subjected to ultrasound-assisted extraction (n = 4), as reported in Section 2.4.

The obtained solutions were concentrated 10 times and injected twice in the HPLC-DAD system under the chromatographic conditions described in Section 2.9. The % recovery (reported in Table S7) was obtained using the following formula:

% Recovery = [(A post-UAE/A pre-UAE)] × 100

A pre-UAE: peak area of standard spiked in the sample, obtained before UAE.
A post-UAE: peak area of standard spiked in the sample obtained after UAE.

*Specificity*. The method specificity was determined using three vegetal matrix samples and comparing the chromatograms obtained after injecting the non-spiked and spiked samples, respectively. Moreover, each sample analysis was followed by a double solvent injection. Nosignal at polyphenol retention times demonstrated the absence of any carry-over effect.

### 2.7. TPC Assay

The phenolic content of the extracts was measured following the procedure described by Redmile-Gordon et al. [23]. The method involves the preparation of two reagents, Reagent A (reacts with proteins to subtract their interference) and Reagent B (not reactive with proteins; it allows for measuring polyphenols), composed of three different stock solutions: (1) 3.5 g of CuSO_4_·5H_2_O in 100 mL H_2_O, (2) 7 g of sodium potassium tartrate in 100 mL of H_2_O, and (3) 70 g of Na_2_CO_3_ in 1 L of NaOH 0.35 N solution. The three solutions were combined in sequence in the proportion 1:1:100 (*v*/*v*/*v*) to obtain Reagent A. Reagent B was prepared in the same way except for solution (1) being replaced by deionized water.

The standard calibration curve was prepared with gallic acid as standard (MW: 170.12 g mol^−1^) to obtain solutions ranging from 15.71 to 31.43 μg mL^−1^.

Standard and sample solutions (prepared as described in Section 2.4) were diluted 1:2 with deionized water. All the solutions were prepared in triplicate. Then, 400 μL of standard and sample dilutions were added to 400 μL of Reagent A. The same procedure was also performed for Reagent B. Samples and standards were incubated at room temperature in the dark for 10 min. After 10 min, 400 μL of Folin–Ciocalteu reagent 1:10, prepared immediately before the end of the first incubation (Folin 2N diluted 1:10 with deionized water and stored in the dark), were added to all standards and samples. Then, the reaction was carried out in the dark for 30 min at room temperature prior to measuring the absorbances at a wavelength of 750 nm (λ_max_). The blank was prepared by adding 400 μL of water, 400 μL of Reagent A or Reagent B (depending on the reagent used), and 400 μL of Folin–Ciocalteu reagent 1:10.

The absorbance values measured at 750 nm (λ_max_) for Reagent A (Abs_A_) and Reagent B (Abs_B_) were used to calculate the absorbance of proteins and the absorbance of polyphenols following equations reported in the literature [23]:

Abs_proteins_ = 1.25 × (Abs_A_ − Abs_B_)

Abs_polyphenols_ = Abs_B_ − 0.2 × (Abs_proteins_)

The Abs_polyphenols_ was used to determine the TPC values, expressed in mmol of gallic acid equivalents (GAE) 100 g^−1^ of DW of lyophilized sample powder.

### 2.8. TAS—ABTS^•+^ Radical Cation Scavenging Activity

TAS was determined through the Trolox Equivalent Antioxidant Capacity (TEAC) assay by spectrophotometric measurement of the ABTS^•+^ radical cation [24]. A solution of ABTS salt (7 mM) in water and a solution of potassium persulfate (2.45 mM) in water were prepared via sonication for 5 min. Then, the ABTS stock solution (A) and the potassium persulfate solution (B) were combined in a ratio of 2:1 (*v*/*v*) to generate ABTS^•+^ radical cation (C). ABTS oxidation was completed in 8 h via incubation in the dark at room temperature. Before measurement, the C solution was diluted 1:3 (*v*/*v*) in ethanol to obtain a working solution (D) with an absorbance of about 0.7 at 734 nm (λ_max_). A calibration curve was established using Trolox as a reference antioxidant, dissolving and diluting it in ethanol to obtain final concentrations in the range of 0.74–23.73 μM.

The D Solution, Trolox solutions, and samples (prepared in triplicate as described in Section 2.4) were equilibrated at 30 °C using the thermomixer. Then, the reaction was carried out directly in the cuvette, in the dark, by adding 10 μL of each Trolox solution and samples to 1.0 mL of solution D (Abs_734nm_ = 0.712), then mixing and waiting 1 min to measure the absorbances at 734 nm. Ethanol was used as blank.

Once all absorbances at 734 nm were registered, the ΔAbs and the percentage of inhibition (I%) of ABTS^•+^ relative to each Trolox solution and sample were calculated as follows:

ΔAbs_734nm_ = A − A1 (sample or Trolox solution)

and

I% = [(ΔAbs_734nm_)/A] × 100;
A: control absorbance (ABTS^•+^);
A1: sample absorbance.

The TAS was expressed as mmol Trolox 100 g^−1^ of dry weight (DW) of lyophilized sample powder.

### 2.9. Determination of the Protein Content by the Kjeldahl Method

The Kjeldahl method was conducted following the guidelines of the Association of Official Agricultural Chemists International (AOAC) [25]. About 1 g of agri-food sample powder lyophilized was hydrolyzed with 15 mL of concentrated sulfuric acid containing 2 copper catalysts at 420 °C for 2 h. After heating, H_2_O was added before proceeding with the neutralization and titration. The total nitrogen shared in the samples was multiplied by 6.25 as the conversion factor, as reported by the protocol.

### 2.10. Statistical Evaluation

All results were expressed as the mean ± SD of 3 independent experiments. Statistical analyses were performed using ordinary one-way ANOVA and Sidak’s multiple comparison tests. The statistical software GraphPad 10.0 version (GraphPad Prism, San Diego, CA, USA) was used, and *p*-values < 0.05 were considered statistically significant.

## 3. Results and Discussion

### 3.1. Gravimetric Determination—Extract Yield (%) by UAE

**Figure 1:**
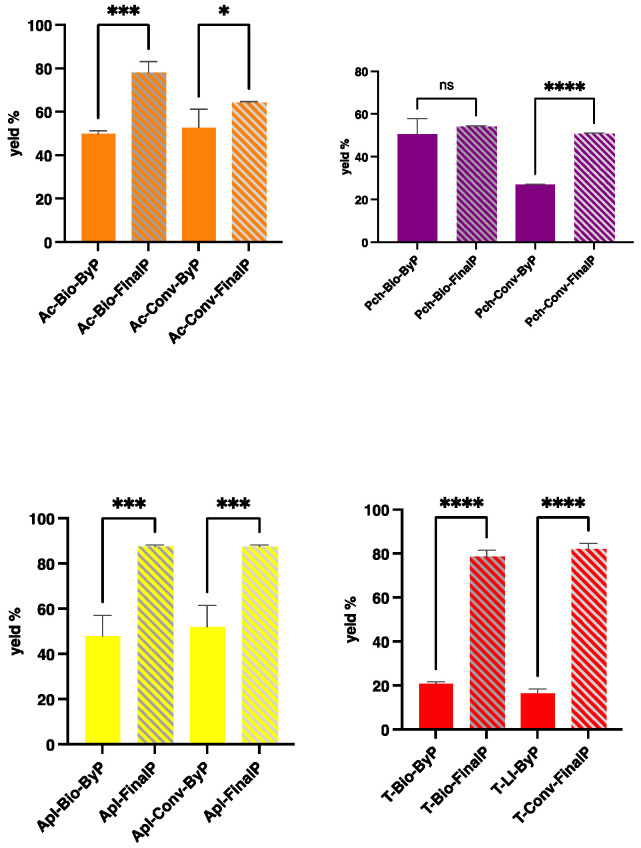
The yield (%) values of the extracts obtained from fruit by-products (ByP) and final products (FinalP) after ultrasound-assisted extraction (UAE). The results are the mean value of 3 extractions ± SD of the same sample. **** *p* < 0.0001; *** *p* < 0.005 * *p* < 0.05. ns = not significant.

The graph reported in Figure 1 compares the extract yield (%) values obtained from fruit by-products and the final products. The findings highlight the quantity of extracts obtained from the by-products being significatively lower than that of their respective final products, except for peach bio by-product and peach bio final products, but they still hold value for further valorization. On the other hand, the juice final products were expected to have a higher extractable portion than their by-products. In particular, according to the waste amount obtained from the different productions, the by-product extraction yields are slightly lower than their relative final products in the case of apricots and peaches; on the contrary, the Apl-FinalP yield accounts for almost 90%. Nonetheless, the lower value obtained for Pch-Conv-ByP is quite surprising and could be ascribed to different starting sample compositions. Regarding tomatoes, the extractable portions obtained from the by-products were expected to be significantly lower than those obtained from the final products. Indeed, the tomato by-products are composed of the peels and seeds of the tomato (Figure S4), and no pulp mass remains in the waste, unlike the other fruits.

### 3.2. Characterization of Agri-Food Samples by HPLC-DAD

**Figure 2:**
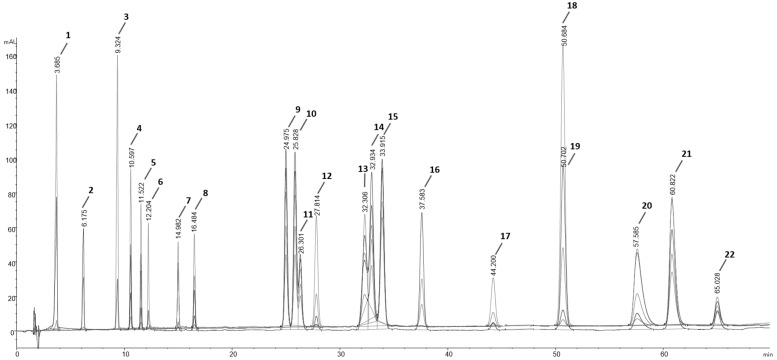
The chromatographic and spectrophotometric analysis of the standard polyphenolic mixture. The overlaid chromatograms were registered at λ = 280 nm; λ = 320 nm; λ = 370 nm; λ = 360 nm; λ = 250 nm; 1 = gallic acid; 2 = protocatechuic acid; 3 = (+)-catechin; 4 = chlorogenic acid; 5 = caffeic acid; 6 = (−)-epicatechin; 7 = p-coumaric acid; 8 = ferulic acid; 9 = hyperoside; 10 = isoquercitrin; 11 = (+)-rutin trihydrate; 12 = phloridzin; 13 = trans-cinnamic acid; 14 = quercitrin; 15 = myricetin; 16 = daidzein; 17 = naringenin; 18 = hesperetin; 19 = genistein; 20 = naringenin chalcone; 21 = kaempferol; 22 = apigenin. The retention time for each compound, the concentrations applied for the chromatographic analysis of the mixture, and the λ_max_ are reported in Table S2.

To determine the polyphenolic content profile of each sample, an HPLC-DAD method was optimized and validated. A mixture containing 22 standard compounds was used to optimize the chromatographic conditions. The chromatographic separation of the reference standard mixture is reported in Figure 2. The HPLC analysis carried out by applying the conditions reported in Section 2.9 allowed to separate and identify all 22 compounds in less than 70 min. Even though hesperetin (peak 18) and genistein (peak 19) were overlaid, it was possible to quantify them since both compounds have different λ_max_ (280 nm and 370 nm, respectively). The chromatographic method was then validated in terms of sensitivity, specificity, linearity, precision, and recovery (data reported in the Supplementary Materials Tables S4–S7). According to the results, the method was found sensitive, with a LoD value ranging between 0.2–16.5 µg mL^−1^ and LoQ values ranging between 0.6–49.9 µg mL^−1^; the precision assessed both intra and inter-day showed an average value of 2.5% and 3.5%, respectively; the optimal linearity was confirmed by an R^2^ value higher than 0.9994 for all the standard compounds; and the recovery showed values in the 90.54–104.44% range, confirming the method being suitable for the quantitative analysis of polyphenols in the samples.

#### 3.2.1. HPLC-DAD Quali/Quantitative Analysis

In the first step of sample characterization, the qualitative analysis was carried out by comparing the retention times evaluated for the standard compounds during the analysis of the mixture to those of the chromatographic peaks revealed for samples chromatograms. The identification was further assessed by overlapping the UV-Vis absorption spectrum (range 210–500 nm) of each peak in the samples acquired at the maximum absorption wavelength with those of the standard compounds. Table S2 shows all the UV-Vis absorption spectra registered for each reference standard, which are characteristic of each phenolic subclass. It is important to observe that many compounds belonging to the same class share similar UV-Vis spectra profiles (as shown in Figure S17). These peculiar spectral features were used to classify unknown substances into phenolic subclasses and identify them as derivatives of some phenolic standards analyzed [22].

Full accounts of retention times of the peaks in all samples analyzed are reported in the Supplementary Materials (Tables S8–S14). The chromatograms obtained for each sample are also reported in Figures S5 and S6 (peach samples), Figures S7 and S8 (apricot samples), Figures S9 and S10 (tomato samples), and Figure S11 (apple samples).

The qualitative analysis was followed by the quantitative one. The latter was carried out by applying the linear regression equation obtained for each standard and interpolating the corresponding peak area of each substance or derivative found in the samples (Table S18).

By combining the information obtained both from the qualitative and quantitative analysis, it was possible to depict a profile of the polyphenolic content of each sample analyzed. Indeed, the comparison of the polyphenolic composition of the corresponding fruit by-product and final product is necessary to reach the main purpose of this work, which is to define the possibility of using the by-products as a source of bioactive ingredients.

##### Apple Products

For apple samples, the quantitative phenolic characterization (Figure S13) shows that phloridzin is the polyphenol retained in the highest amount in apple by-products, comparable to the quantity found in Apl-FinalP (28.80 ± 2.86 mg 100 g^−1^ DW). Interestingly, flavonols (hyperoside, quercitrin, myricetin, and rutin) are retained even in higher amounts in the Apl-Bio-ByP than in the Apl-Conv-ByP and Apl-FinalP, where hyperoside stands as the main flavonol present in the highest amount (19.38 ± 1.74 mg 100 g^−1^ DW), even when compared to all fruit samples studied, followed by quercitrin at 17.09 ± 5.77 × 10^−2^ mg 100 g^−1^ DW. Compared to peach and apricot samples, apple samples showed a lower amount of chlorogenic acid in their composition.

**Figure 3:**
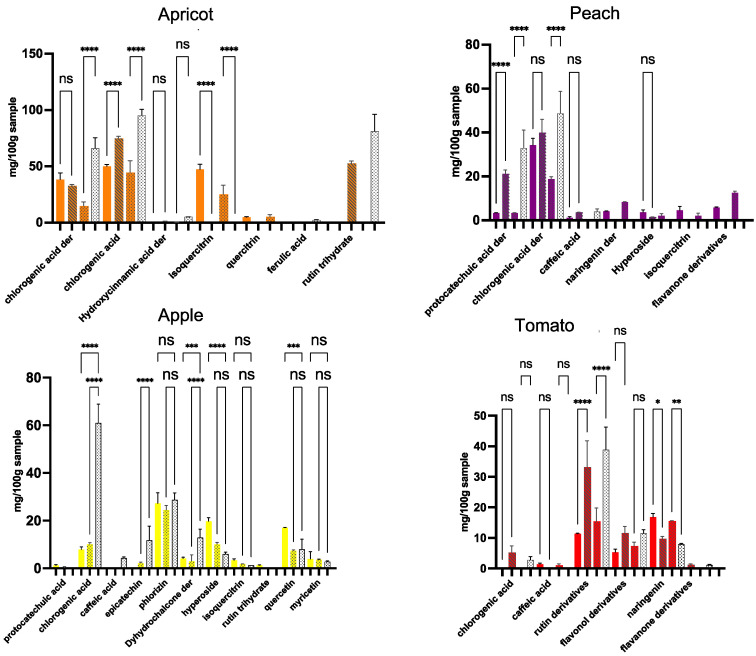
Bar charts reporting the phenolic profile of the studied samples. The amount of phenolic acids and flavonoids, characterized in both the biological (colored columns) and conventional (colored/dotted columns) by-products, are compared with those found in the biological final products (colored/ribbed columns) and the conventional final ones (uncolored/dotted columns), respectively. The results are the mean value carried out from 3 analyses ± SD of the same sample. **** *p* < 0.0001; *** *p* < 0.005; ** *p* < 0.01; * *p* < 0.05. ns = not significant.

According to Figure S11, which reports the comparison in qualitative analysis between Apl-Bio-ByP and Apl-FinalP, the following compounds, protocatechuic acid, chlorogenic acid, p-coumaric acid, hyperoside, isoquercitrin, phloridzin, quercitrin, and myricetin, are present in both the samples. The presence of phloridzin (peak 12) and other hypothetical glycosidic derivatives (peaks 29), which elute in a shorter time, is the most important feature of apples, both in the by-product and in the final product. The phenolic class distribution was found to be more heterogeneous in apple samples (Figure 3), showing a predominance of flavonols, dihydrochalcones, and hydroxycinnamic acids.

##### Peach Products

The chromatographic analysis of Pch-Bio-ByP and Pch-Bio-FinalP followed by the UV-Vis spectra analysis of identified peaks and families’ member peaks (Figure S5) allowed us to define the relative distribution of polyphenols of different classes in the samples. Several phenolic compounds were unambiguously identified, such as chlorogenic and caffeic acids, hyperoside, naringenin, and isoquercitrin (Figure S5). However, many unassigned peaks (also with high intensity, i.e., 23, 24, 27) were still classified as members of polyphenol families due to their UV-Vis absorption spectra similarity in comparison to standards. Peak 24 in both chromatograms shows the same UV-Vis spectra of chlorogenic acid (Figure S5, a_2_; b_2_) eluting at an earlier retention time (7.302 min in Pch-Bio-ByP and 7.296 min in Pch-Bio-FinalP). According to the literature, this peak may be neochlorogenic acid (NCHA), an isomer of chlorogenic acid, present at high concentrations in peaches [26]. The study shows an elution time for NCHA at around 7 min and chlorogenic acid at around 10 min (HPLC-DAD) [26], which supports the idea that this compound is NCHA. The results from the quali-quantitative phenolic characterization of peach samples (Figure S12) show that peach by-products can retain significant amounts of chlorogenic acid and its derivative. Pch-Bio-ByP was the peach by-product with the highest concentrations of such hydroxycinnamic acids (34.29 ± 9.71 × 10^−1^ and 34.26 ± 3.15 mg 100 g^−1^ DW, respectively). Interestingly, some flavonoids, such as isoquercitrin and flavanone derivatives, were only detectable in the by-product samples, which could mean that after the juice pressing, these flavonoids can be further concentrated and retained.

##### Apricot Products

Similarly, the quali-quantitative phenolic characterization of apricot samples (Figure S14) shows that apricot by-products tend to retain even higher amounts of chlorogenic acid and its derivative NCHA than those observed for peach samples, where the Ac-Bio-ByP shows the highest concentrations of such compounds (49.95 ± 1.51 and 38.30 ± 5.92 mg 100 g^−1^ DW, respectively) when compared to all the by-products studied, as reported in Figure 3. Regarding flavonoids, the results evidence that isoquercitrin was significantly retained in apricot by-products, exhibiting the highest amounts (47.37 ± 4.63 mg 100 g^−1^ DW for Ac-Bio-Byp and 25.07 ± 8.20 mg 100 g^−1^ DW for Ac-Conv-ByP) when compared to the other fruit by-products under study (Figure 3). Interestingly, quercitrin proved to be present at significative levels (4.80 ± 4.39 × 10^−1^ mg 100 g^−1^ DW for Ac-Bio-Byp and 5.09 ± 1.76 mg 100 g^−1^ DW for Ac-Conv-ByP) in apricot by-products as well. In this sense, as displayed in Figure S7, the phenolic profile of apricot samples is mainly characterized by a high fraction of hydroxycinnamic acids (mainly comprising chlorogenic acid and its derivative), followed by flavonols, whose main compound was found to be isoquercitrin.

##### Tomato Products

On the other hand, the quali-quantitative phenolic characterization of tomato samples (Figure S15) showed that naringenin was the main polyphenol existing in higher amounts, even when compared to all fruit samples studied (Figure 3). Particularly, tomato by-products were found to retain naringenin in significant amounts (16.83 ± 1.71 mg 100 g^−1^ DW for T-Bio-ByP and 15.57 ± 2.41 × 10^−2^ mg 100 g^−1^ DW for T-LI-ByP), even higher than in their final products, as well as rutin (11.31 ± 2.71 × 10^−1^ mg 100 g^−1^ DW for T-Bio-ByP and 15.37 ± 4.44 mg 100 g^−1^ DW for T-LI-ByP) and other unidentified flavonol derivatives. Nonetheless, tomato samples proved to be overall the poorest source of polyphenols when compared to the other fruit samples (Figure 3), which correlates to the results obtained in the TPC and TAS analysis. Tomato samples highlight a distinct qualitative profile, in which flavonoids (flavanones and flavonols specifically) are widely distributed in samples (Figure S9). Furthermore, as in peach samples, naringenin and its derivative were also detected in tomato final product samples.

The obtained results can be summarized as follows. All the peach by-products show chlorogenic acid and its derivatives as the most abundant compounds. The same compounds in the ByP are less abundant than in the FinalP. It is worth mentioning that the concentration of chlorogenic acid derivative in Pch-Bio-ByP is higher than that revealed for the analyses of Pch-Conv-ByP, and it is almost similar to that of Pch-Bio-FinalP. Interestingly, the characterized flavonoid derivatives are even more abundant in ByP than in FinalP. Similar results were obtained for apricot products, for which a huge amount of isoquercitrin was found in both Ac-ByP. This interesting trend was also revealed for Apl-ByP. In these samples, the amount of hyperoside, quercitrin, and myricetin is higher than that found in the Apl-FinalP. On the contrary, the amount of chlorogenic acid contained in this latter is six times higher than that shown for Apl-ByP. On the contrary, the tomato by-products present a very low amount of chlorogenic acid. The interest in T-ByP is indeed confirmed by a consistent type and concentration of flavonoid derivatives. In particular, the amount of naringenin in T-ByP is even higher than that found in T-FinalP.

### 3.3. TPC

The calibration curve of standard gallic acid (15.71–31.43 µg mL^−1^), obtained by following the procedure reported in Section 2.7, showed a linear equation (y = 0.0512x − 0.1882; LoD = 5.07 µg mL^−1^; LoQ = 15.35 µg mL^−1^) with an R^2^ = 0.997 and was used to determine the TPC of all the samples.

**Figure 4:**
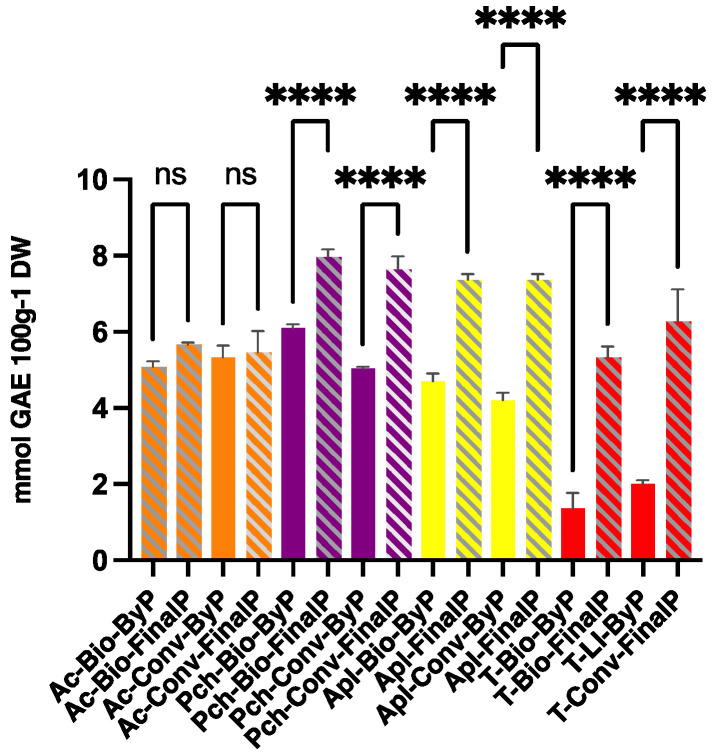
Total phenolic content (TPC) values of extracts obtained from ByP and FinalP via ultrasound-assisted extraction (UAE). The results are the mean value of 3 extractions ± SD of the same sample. **** *p* < 0.0001. ns = not significant.

Figure 4 shows the TPC values expressed as mmol GAE 100 g^−1^ DW obtained for all the samples.

The TPC for those by-products with a high pulp content, such as apricot and peach by-products, was very high and comparable to those obtained for their final products. In particular, in the case of apricots, the TPC values of all the samples are very close to each other, indicating that the amounts of polyphenols remaining in the by-products are comparable to that present in the final products. As expected for apple samples, the by-products show lower but valuable TPC than the final products. For tomato samples, the TPC values of the by-products are significantly lower than those of their final products, which is explained, as said before, by the composition of by-products and by the low extraction yield observed in the gravimetric determination (Section 3.1).

### 3.4. Total Antioxidant Status Assay (TAS)

The calibration curve of Trolox (0.74–23.73 µM) was performed to obtain a linear equation (y = 3.8189x − 3.3127; LoD = 0.04 µM; LoQ = 0.11 µM; R^2^ = 0.9935) for evaluating the antioxidant activity of the samples, which was expressed as mmol Trolox 100 g^−1^ DW.

**Figure 5:**
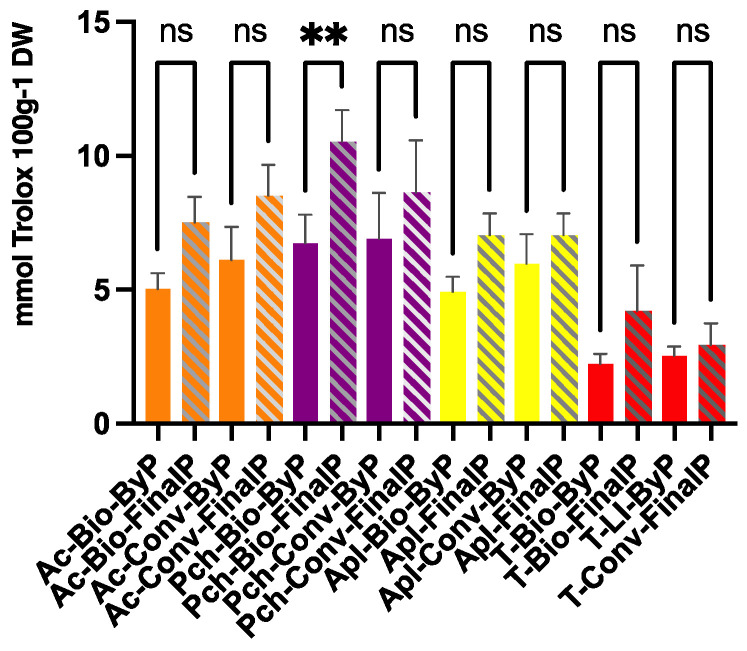
Total antioxidant status (TAS) values of extracts obtained from fruit ByP and FinalP via UAE. The results are the mean value of 3 extractions ± SD of the same sample. ** *p* < 0.005. ns = not significant.

Figure 5 shows the TAS values for all the samples obtained following the methodology described in Section 2.8. Overall, the results showed that final products display a similar TAS value compared to that of the by-products, except for Pch-Bio-Ps. This is an interesting result since the TPC levels evaluated for both the ByPs and FinalPs were found to be significatively different for all the fruit samples except for apricot products.

In general, it can be noted that peach samples tend to have the highest antioxidant activity, followed by apricot ones. For almost all the samples, it is interesting to note that the by-products exhibit a TAS value comparable to that of the final products. This feature observed for apple by-products is in contrast to the previous results, where Apl-FinalP revealed the highest difference between the by-product and final product related to the extract yield (%) and to TPC values. The unexpectedly high TAS value obtained for these by-products may be explained by the composition of such extracts. Indeed, despite Pch-Bio, Pch-Conv, Apl-Bio, Apl-Conv, T-Bio, and T-LI by-products having lower TPC values than their final products, they may contain a remarkable percentage of phenolic compounds, such as flavonoids, whose chemical structure is linked to a higher capacity of scavenging free radicals [27,28].

**Figure 6:**
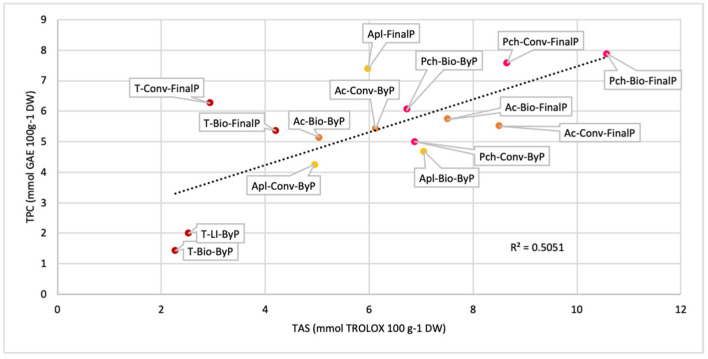
Correlation between TPC and the TAS values of fruit byP and finalP.

**Figure 7:**
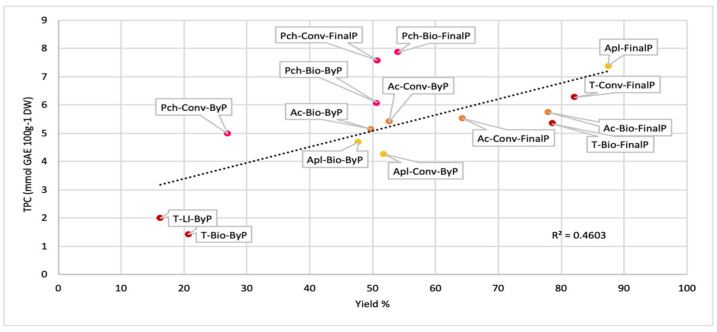
Correlation between the extract yield (%) and the TPC values of fruit byP and FinalP.

The analysis of the graph reported in Figure 6, in which TPC and TAS values are correlated, indicates that a higher content of phenolic compounds might not be necessarily related to a higher antioxidant activity. This aspect confirms the importance of characterizing the bioactive compounds since the type and the nature of the polyphenol contained in the sample might influence the TAS values greatly. Other important considerations that can help in considering by-products for their valorization have to be conducted on the results obtained by correlating the TPC values to the extract yield (%) (Figure 7). According to the results previously discussed, a higher yield of extraction does not necessarily relate to a proportional increase in the content of phenolic compounds. This observation is more evident for the peach and apricot samples studied, whereas for apple and tomato samples, a higher correlation is visible between by-products and final products.

The TPC value for each group of samples was also correlated to the sum of the individual phenolic compounds (SPC) determined by HPLC. The graphs reported in Figure S16 suggest a good correlation between the TPC and the Sum of the individual quantities of Phenolic Compounds (SPC) values except for apricot (Ac) samples. A TPC assay is indeed a quick method, which can be used for a preliminary indication of the polyphenol yield when optimizing the extraction, but many chemically similar compounds interfere with the test results [29]. Therefore, the HPLC method is strictly required for an accurate quantification of polyphenols. The difference in TPC and SPC can be ascribed to all the compounds that interfere in the TPC assay as well as to the types and levels of interferents, which depend on the natural source of the extract [30].

This evidence allows for the characterization of the samples from both a chemical and functional point of view, thus optimizing the reuse of the studied by-products in a circular economy prospect.

### 3.5. Protein Content Evaluation

**Figure 8:**
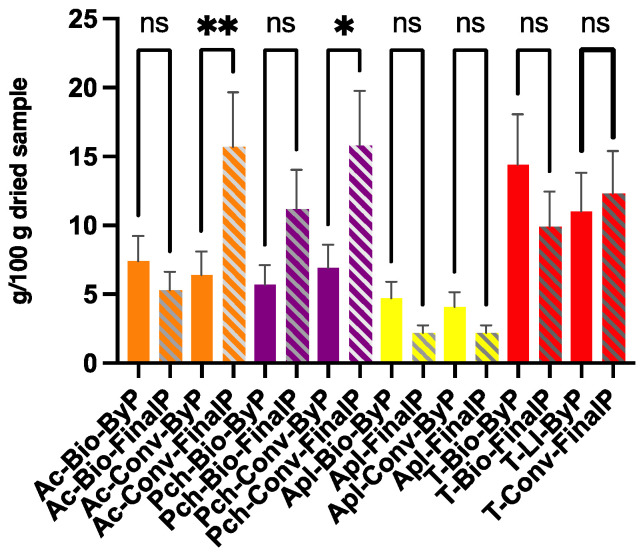
Protein content obtained from fruit ByP and FinalP by the Kjeldahl method. The results are the mean value of 2 experiments ± SD. ** *p* < 0.01; * *p* < 0.05. ns = not significant.

The protein content for each sample has been evaluated by the Kjeldahl method. The obtained results are reported in Figure 8. The values for protein content are expressed as g 100 g^−1^ DW. It is possible to notice that most of fruit by-products exhibits a protein content comparable to that of their final products.

Interestingly, both the apple and tomato by-products showed a very high protein content comparable to or greater than those of their final products. Indeed, the tomato by-products’ protein content was two to three times higher than that of the other fruit by-products. These results can be correlated to the fact that both apple and tomato by-product composition is primarily made of their corresponding seeds, which have been described as rich sources of proteins, accounting for up to 49.55% and 32% of their nutritional value, respectively [10].

Apricot and peach bio by-products have similar protein content, representing a lower or similar value than that of their final product counterpart. In general, the fact that conventional apricot and peach final products show the highest protein contents can be explained by considering conventional farming as associated with the use of pesticides, which can also contain nitrogen that can interfere with this method. However, even when obtained through conventional farming, the final products under study did not show any significant pesticide level; therefore, the results can be ascribed to the actual protein contents.

### 3.6. Valorization of By-Products under Study

Plant polyphenols have been studied for their numerous benefits on human health. Their activities are especially related to the treatment and prevention of several chronic diseases [31]. Moreover, their beneficial interactions with the gut microbiota and prebiotic modulation have been assessed recently as well [32].

From a circular economy perspective, the possibility of keeping materials in circulation has pointed out the opportunity to valorize wastes from the agri-food industry. Studies carried out with this aim have evidenced the recovery of phenolic compounds from by-products, showing the potential therapeutic applications in the nutraceutical field due to their benefits as antiproliferative, antioxidant, and anti-inflammatory agents [33,34].

To optimize the reuse of fruit by-products as alternative sources of polyphenols, we carried out this study that ultimately confirmed that the investigated industrial wastes can retain significant amounts of several phenolic subclasses present in their traditional fruit counterparts (final products), as summarized in Figure 3. In particular, the quali-quantitative analyses evidenced phloridzin as a distinctive polyphenol found in apples [35]. It was shown to be equally retained in the apple by-product extracts studied when compared to their respective final product (apple juice). This supposes the possibility of using these by-products in the recovery of this valuable compound. Indeed, extracts rich in phloridzin could be promising candidates to develop formulations destined for managing metabolic disorders, such as diabetes, due to their reported hypoglycemic activity [36,37]. Apple by-products under study were shown to be rich in different phenolic subclasses. Particularly, the biological apple by-product was found to concentrate higher quantities of flavonols after juice processing. Among them, hyperoside (quercetin-3-O-galactoside) and quercitrin (quercetin 3-rhamnoside) add greater value to this by-product due to the well-known structurally related antioxidant/antiradical profile of quercetin and its derivatives [38,39,40]. This could further explain the higher TAS value obtained for Apl-Bio-ByP than that expected regardless of its low TPC value.

The results also depicted a significant antioxidant activity through the TAS assay for peach and apricot by-products, seemingly related to the high amounts of chlorogenic acids quantified in these samples. Indeed, chlorogenic acids were found to be the main phenolic compounds in peach and apricot by-products, remaining in considerable amounts also after juice processing. This result could open up the opportunity to valorize the extracts from these by-products and take advantage of the antioxidant activity related to these compounds [41]. At the same time, the high and unique presence of the flavonol isoquercitrin (quercetin-3-O-β-d-glucopyranoside), found exclusively in the apricot by-products, could increase the value of extracts obtained from these upcycled materials even more.

Regarding tomato by-product extracts, although they showed the lowest TPC and TAS values, the quali-quantitative analyses of their composition, obtained by HPLC-DAD, evidenced these materials as valuable sources of flavonoids too. In particular, the concentration of the flavanone naringenin in these waste materials surpassed that found in their traditional tomato counterparts (final products). In this sense, tomato by-products can be considered alternative sources of naringenin. That is a significant aspect since naringenin is a polyphenol with promising supplement applications for cardiovascular protection [42]. Nonetheless, the main valorization of tomato by-products might be directed to the extraction of proteins since these materials were found to contain the highest values of proteins among fruit by-products. Then high-quality proteins could be recovered and/or used to produce supplements rich in bioactive peptides [10].

All these results give the possibility of designing and carrying out new strategies for the further valorization of biomass from the agri-food industry in a circular economy approach. The characterized residues may also retain other chemical compounds of interest, such as carbohydrates, carotenoids (ß-carotene and lycopene), and lipids (especially in tomato by-products [10]), which may be exploited for added health-promoting properties.

## 4. Conclusions

All primary analyses carried out in the present research allowed us to verify the fruit waste materials that are rich in bioactive molecules. The results obtained by the conducted experiments indeed evidenced the opportunity to derive from the studied by-products ingredients suitable for the formulation of both nutraceuticals and dietary supplements. Indeed, the analyzed fruit by-products proved them as rich sources of polyphenols of heterogeneous chemistry. These compounds, endowed with the ability to act as bioactive and antioxidant agents, were found in significant concentrations in such materials. The HPLC-DAD method applied to the aim of characterizing samples in terms of polyphenol content resulted easy to use, fast, and applicable to a wide range of fruit samples with different chemical complexity. The HPLC-DAD analysis confirmed that most of the fruit by-products contained valuable polyphenols in a fair amount in comparison to their industrial final product, retaining the same compounds after juice processing, or even enhancing the presence of other polyphenols. Indeed, apricot, apple, and peach by-products presented significant amounts of polyphenols and a wide diversity of phenolic subclasses comprising several hydroxycinnamic acids as well as different types of flavonoids. On the contrary, tomato by-products proved to be greater sources of proteins, with concentrations comparable or greater to those obtained from the final product.

Results reported in this study represent a first step for promoting a circular economy strategy where the agri-food sector could valorize such waste materials as potential candidates to develop sustainable novel ingredients as alternatives to traditional ones for exploitation in the nutraceutical and food supplement fields. The results obtained by the reported experiments showed that the studied by-product extracts could be active ingredients suitable for the formulation of both nutraceuticals and dietary supplements. Active ingredients used in nutraceutical products could treat or reduce the risk of counteracting some diseases, while the ones present in the dietary supplements should compensate deficiencies in micro- or macronutrients [43]. Therefore, it would be reasonable to propose the application of polyphenol extracts for nutraceutical formulations in light of their antioxidant properties and of proteins for the food supplements preparation. What is more is that the possibility to apply well-characterized ingredients from by-products for nutraceutical or food supplement preparations could avoid some adulteration and contamination issues, thus ensuring, besides efficacy, also the safety of these products. In order to assess the in vivo efficacy of the extracts, the metabolic pattern of the most promising samples will be studied. Further researchs may concern the replacement of methanol and acetone with greener and safer alternatives, such as water, ethanol, and even novel deep eutectic solvents (DES) that may lead to extracts that are “ready to use” for different purposes. Additional studies focused on the valorization of the biomass that remains after the extraction of polyphenols represents an engaging opportunity for further development.

## Figures and Tables

**Table 1:** Agri-food samples under study.

Sample		Acronym
Apricot	Biological by-product	Ac-Bio-ByP
	Conventional by-product	Ac-Conv-ByP
	Biological final product	Ac-Bio-FinalP
	Conventional final product	Ac-Conv-FinalP
Peach	Biological by-product	Pch-Bio-ByP
	Conventional by-product	Pch-Conv-ByP
	Biological final product	Pch-Bio-FinalP
	Conventional final product	Pch-Conv-FinalP
Apple	Biological by-product	Apl-Bio-ByP
	Conventional by-product	Apl-Conv-ByP
	Apple final product	Apl-FinalP
Tomato	Biological peels	T-Bio-ByP
	LI* peels	T-LI-ByP
	Biological final product	T-Bio-FinalP
	Conventional final product	T-Conv-FinalP

Lotta integrata* is a crop defense practice that aims to reduce pesticide use through the implementation of multiple measures. It is the intermedium between conventional and biological methodologies.

## Data Availability

Data is contained within the article and Supplementary Materials.

## Supplementary Materials

The following supporting information can be downloaded at: https://www.mdpi.com/article/10.3390/antiox13050604/s1.

## Abbreviations

UAE	Ultrasound-assisted extraction.
HPLC-DAD	High-performance liquid chromatography–diode array detector.
TPC	Total phenolic content.
TAS	Total antioxidant status.
TEAC	Trolox equivalent antioxidant capacity.
ByP	By-products.
FinalP	Final products.
Ac-Bio-ByP	Apricot biological by-product.
Ac-Conv-ByP	Apricot conventional by-product.
Ac-Bio-FinalP	Apricot biological final product.
Ac-Conv-FinalP	Apricot conventional final product.
Pch-Bio-ByP	Peach biological by-product.
Pch-Conv-ByP	Peach conventional by-product.
Pch-Bio-FinalP	Peach biological final product.
Pch-Conv-FinalP	Peach conventional final product.
Apl-Bio-ByP	Apple biological by-product.
Apl-Conv-ByP	Apple conventional by-product.
Apl-FinalP	Apple final product.
T-Bio-ByP	Tomato biological peels.
LI	Lotta integrata.
T-LI-ByP	Tomato lotta integrata biological peels.
T-Bio-FinalP	Tomato biological final product.
T-Conv-FinalP	Tomato conventional final product.
DW	Dry weight.
LoD	Limit of detection.
LoQ	Limit of quantification.
SS	Stock solutions.
AOAC	Association of Official Agricultural Chemists International.
NCHA	Neochlorogenic acid.
SPC	Sum of the individual phenolic compounds.
DES	Deep Eutectic Solvents.
