# Supplementary material for: Phenolic and Antioxidant Characterization of Fruit By-Products for Their Nutraceuticals and Dietary Supplements Valorization under a Circular Bio-Economy Approach
Source: Antioxidants (Basel). 2024 May 14;13(5):604. doi: 10.3390/antiox13050604 (PMC11118151; doi:10.3390/antiox13050604)
Supplement: Supplementary file 1 [file antioxidants-13-00604-s001.zip › antioxidants-2962532-supplementary.pdf]

Phenolic and Antioxidant Characterization of Fruit By-Products for their Valorization as Nutraceuticals and Dietary Supplements under a Circular Bio-Economy Approach

Figure S1. Apricot samples from juice processing, including the by-product (ByP) known as apricot pomace and the final product (FinalP).

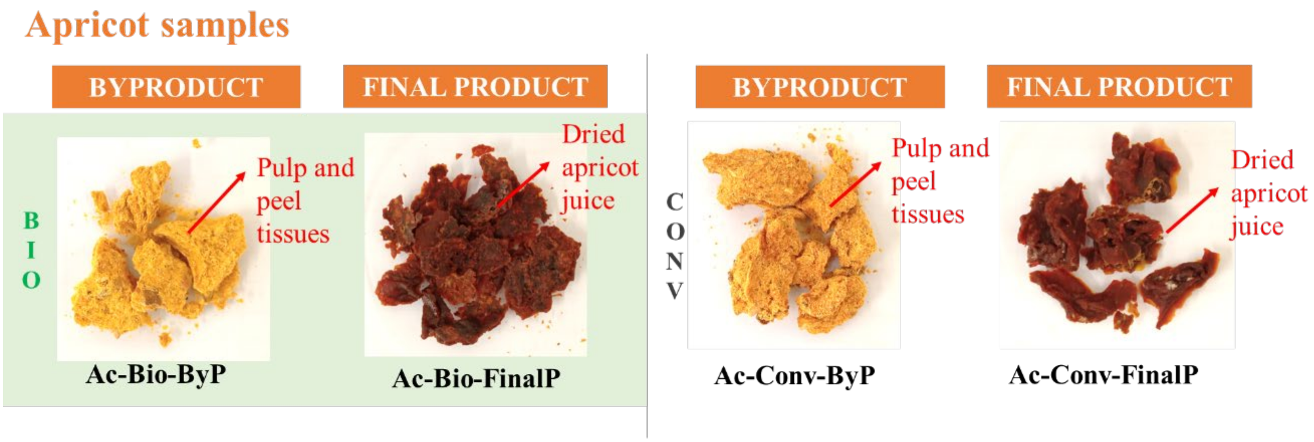

Figure S2. Peach samples from juice processing, including the by-product (ByP) known as peach pomace and the final product (FinalP).

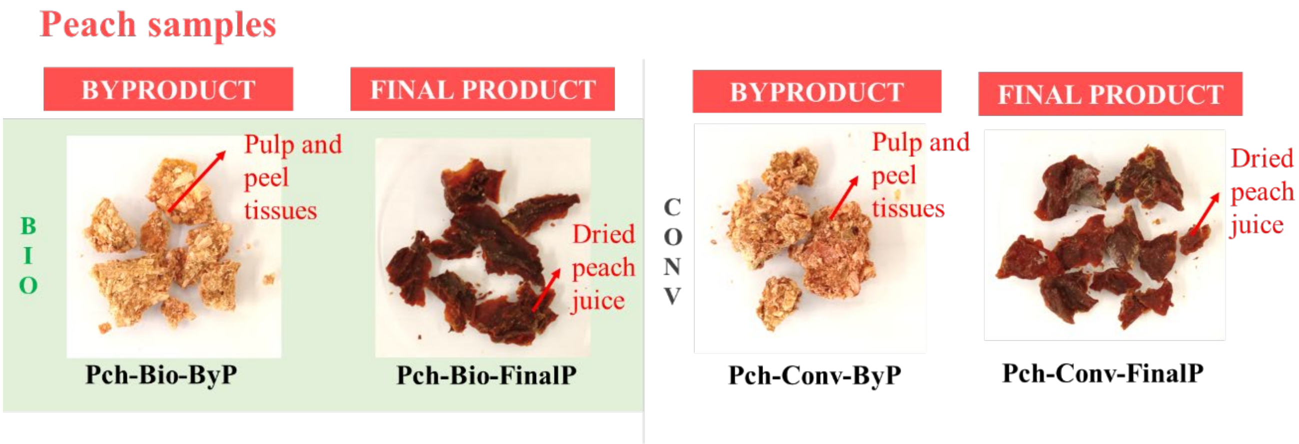

Figure S3. Apple samples from apple juice processing, including the by-product (ByP) known as apple pomace and the final product (FinalP).

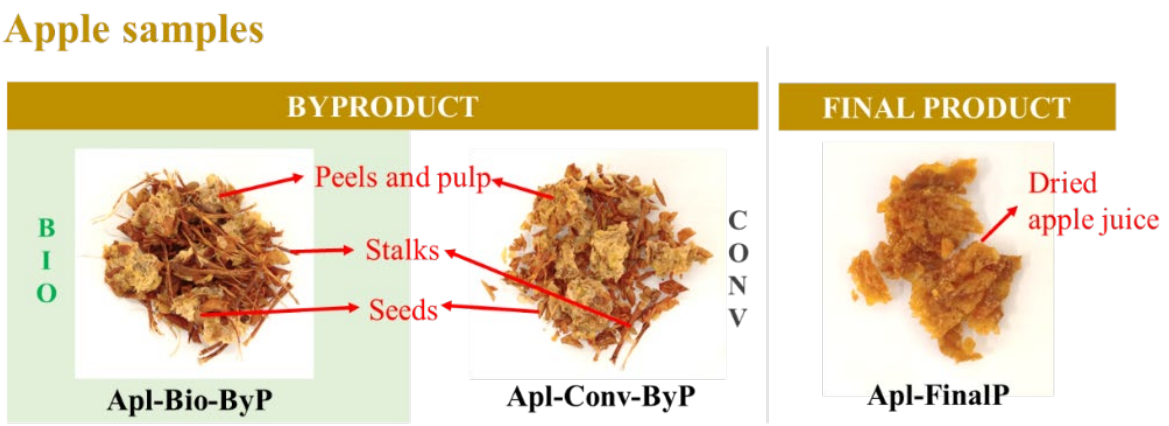

Figure S4. Tomato samples from tomato sauce processing, including the by-product (ByP) and the final product (FinalP).

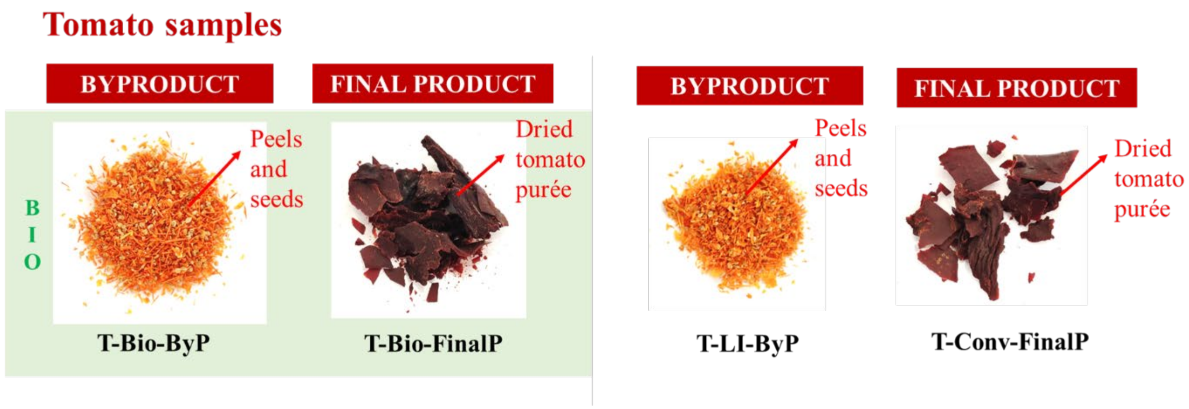

Table S1. Gravimetric determination, total phenolic content, total antioxidant status (measured with the ABTS assay), and protein content of agri-food samples.

Samples analyzed	Gravimetric Determination<br>Yield (%) ±SD<br>(n=3)	Total Phenolic Content (TPC)<br>Average mmol GAE 100g^{-1} DW ±SD<br>(n=3)	Total Antioxidant Status (TAS)<br>Average mmol Trolox 100g^{-1} DW ±SD<br>(n=3)	Kjeldahl method: protein content<br>Average g 100g^{-1} DW ±SD
Ac-Bio-ByP	49.8±7.29E-01	5.14±1.71E-01	5.04±5.58E-01	7.40±1.30E+00
Ac-Conv-ByP	52.6±4.23E+00	5.42±3.22E-01	6.12±1.26E+00	6.40±1.20E+00
Ac-Bio-FinalP	78.0±2.53E+00	5.73±1.18E+00	7.51±9.60E-01	5.30±9.50E-01
Ac-Conv-FinalP	64.3±1.42E-01	5.52±1.34E+00	8.51±1.14E+00	15.70±2.80E+00
Pch-Bio-ByP	50.7±3.57E+00	6.06±4.71E-01	6.73±1.05E+00	5.70±1.00E+00
Pch-Conv-ByP	27.0±4.58E-02	4.99±1.58E-01	6.88±1.69E+00	6.90±1.20E+00
Pch-Bio-FinalP	54.1±2.31E-01	7.87±1.05E+00	10.57±1.17E+00	11.20±2.00E+00
Pch-Conv-FinalP	50.7±1.59E-01	7.56±5.09E-01	8.66±1.93E+00	15.80±2.80E+00
Apl-Bio-ByP	47.7±4.65E+00	4.68±3.34E-01	7.06±7.96E-01	4.70±8.50E-01

Apl-Conv-ByP	51.8±4.81E+00	4.25±4.97E-01	4.95±5.69E-01	4.10±7.40E-01
Apl-FinalP	87.5±3.24E-01	7.38±1.04E+00	5.98±1.10E+00	2.20±4.00E-01
T-Bio-ByP	20.7±9.08E-01	1.42±5.98E-01	2.27±3.65E-01	14.40±2.60E+00
T-LI-ByP	16.3±2.18E+00	1.99±9.35E-02	2.53±3.69E-01	11.00±2.00E+00
T-Bio-FinalP	78.6±2.96E+00	5.35±2.95E-01	4.21±1.66E+00	9.90±1.80E+00
T-Conv-FinalP	82.1±2.55E+00	6.27±8.81E-01	2.94±7.71E-01	12.30±2.20E+00

HPLC-DAD analysis

Table S2. Polyphenol standards used for method optimization and validation.

Nº	Compound	λ_{max} (nm)	Retention Time (min)	Picomoles mL^{-1}	Phenolic subclass	Structure
1	Gallic Acid	280	3.685	1.65E+08	Hydroxybenzoic acid	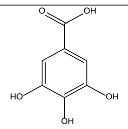
2	Protocatechuic acid	250	6.175	7.30E+07	Hydroxybenzoic acid	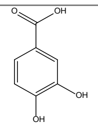
3	(+)-Catechin	280	9.324	2.26E+08	Flavan-3-ol	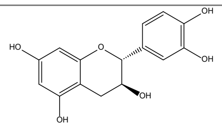
4	Chlorogenic acid	320	10.597	2.65E+07	Hydroxycinnamic acid	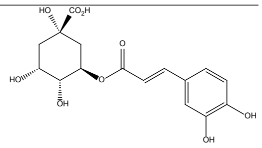
5	Caffeic acid	320	11.522	2.08E+07	Hydroxycinnamic acid	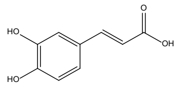
6	(-)-Epicatechin	280	12.204	9.69E+07	Flavan-3-ol	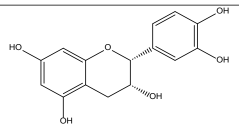
7	p-coumaric acid	320	14.982	1.14E+07	Hydroxycinnamic acid	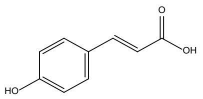

**8**	Ferulic acid	320	16.484	2.90E+07	Hydroxycinnamic acid	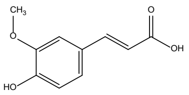
**9**	Hyperoside	250	24.975	8.07E+07	Flavonol	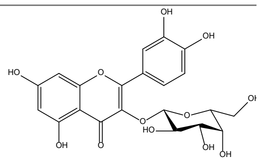
**10**	Isoquercitrin	250	25.828	8.07E+07	Flavonol	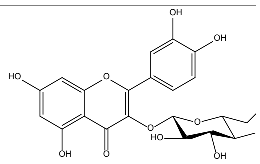
**11**	(+)-Rutin Trihydrate	250	26.301	3.07E+07	Flavonol	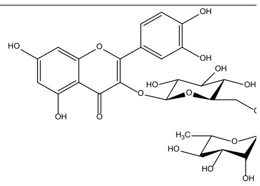
**12**	Phloridzin dihydrate	280	27.814	5.95E+07	Dihydrochalcone	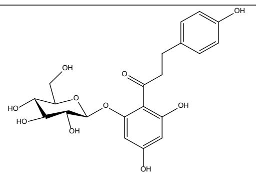
**13**	Trans-cinnamic acid	280	32.306	5.06E+07	Hydroxycinnamic acid	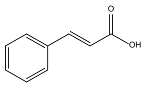
**14**	Quercitrin	250	32.934	1.25E+08	Flavonol	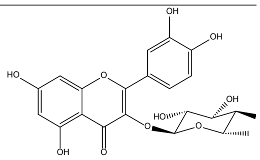
**15**	Myricetin	370	33.915	6.22E+07	Flavonol	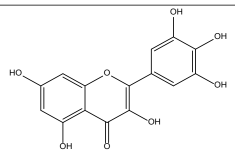
**16**	Daidzein	250	37.583	6.69E+07	Isoflavone	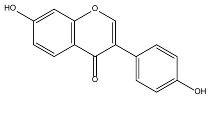
**17**	Naringenin	280	44.200	2.75E+07	Flavanone	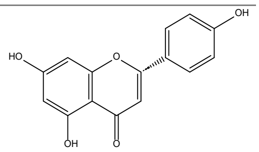

**18**	Genistein	250	50.684	5.40E+07	Isoflavone	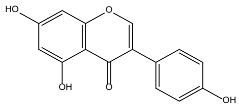
**19**	Hesperetin	280	50.702	9.33E+07	Flavanone	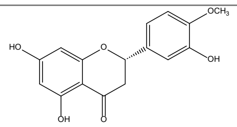
**20**	Naringenin chalcone	370	57.585	6.06E+07	Chalcone	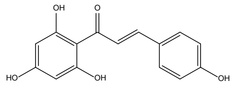
**21**	Kaempferol	370	60.822	4.19E+07	Flavonol	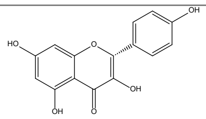
**22**	Apigenin	320	65.028	13.200	Flavone	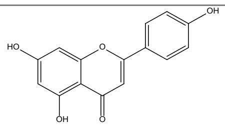

Table S3. Standard concentration in the mixture whose chromatogram is reported in Figure 6.

**Standard**	**Compound**	**Concentration µg mL^{-1}**
1	Gallic Acid	28.125
2	Protocatechuic acid	11.250
3	(+)-Catechin	65.500
4	Chlorogenic acid	9.375
5	Caffeic acid	3.750
6	(-)-Epicatechin	28.125
7	P-coumaric acid	1.875
8	Ferulic acid	5.625
9	Hyperoside	37.500
10	Isoquercitrin	37.500
11	(+)-Rutin Trihydrate	18.750
12	Phloridzin dihydrate	28.125
13	Trans-cinnamic acid	7.500
14	Quercitrin	56.250
15	Myricetin	19.800
16	Daidzein	17.000
17	Naringenin	7.500
18	Genistein	14.580
19	Hesperetin	28.200
20	Naringenin chalcone	16.500
21	Kaempferol	12.000
22	Apigenin	13.200

Table S4. Data concerning calibration curves of polyphenols standards, including LoD and LoQ values.

Std	Compound	λ (nm)	rt (min)	Linear Range µg mL^{-1}	Calibration Curves	R^2	LoD (µg mL^{-1})	LOQ
1	Gallic Acid	280	3.685	31.60-240.00	y = 71.466x + 198.82	0.9994	10.40	31.60
2	Protocatechuic acid	250	6.175	7.00-100.00	y = 44.709x + 49.354	0.9998	2.30	7.00
3	(+)-Catechin	280	9.324	19.40-240.00	y = 19.454x + 52.856	0.9998	6.40	19.40
4	Chlorogenic acid	320	10.597	49.90-300.00	y = 84.437x + 353.85	0.9999	16.50	49.90
5	Caffeic acid	320	11.522	3.60-60.00	y = 127.5x + 74.584	0.9999	1.20	3.60
6	(-)-Epicatechin	280	12.204	10.70-128.00	y = 14.039x + 21.127	0.9997	3.50	10.70
7	P-coumaric acid	320	14.982	0.60-10.00	y = 241.61x + 23.811	0.9999	0.20	0.60
8	Ferulic acid	320	16.484	2.60-32.00	y = 99.2x + 35.838	0.9998	0.90	2.60
9	Hyperoside	250	24.975	7.30-150.00	Y=49.26x + 44.529	0.9999	2.40	7.30
10	Isoquercitrin	250	25.828	2.80-40.00	y = 49.507x + 17.742	0.9998	0.90	2.80
11	(+)-Rutin Trihydrate	250	26.301	5.00-64.00	y = 37.121x + 13.267	0.9998	1.70	5.00
12	Phloridzin dihydrate	280	27.814	7.30-96.00	y = 41.09x + 44.011	0.9998	2.40	7.30
13	Trans-cinnamic acid	280	32.306	1.50-24.00	y = 156.96x + 30.134	0.9998	0.50	1.50
14	Quercitrin	250	32.934	13.30-256.00	y = 31.202x + 64.347	0.9999	4.40	13.30
15	Myricetin	370	33.915	7.50-128.00	y = 118.7x - 39.818	0.9999	2.50	7.50

16	Daidzein	250	37.583	8.50-160.00	y = 111.22x + 142.73	0.9999	2.80	8.50
17	Naringenin	280	44.200	5.20-64.00	y = 64.552x + 39.8	0.9998	1.70	5.20
18	Genistein	250	50.684	7.70-96.00	y = 160.73x + 172.32	0.9998	2.50	7.70
19	Hesperetin	280	50.702	12.60-160.00	y = 114.62x + 213.99	0.9998	4.20	12.60
20	Naringenin chalcone	370	57.585	8.10-96.00	y = 125.18x + 120.48	0.9997	2.70	8.10
21	Kaempferol	370	60.822	5.30-96.00	y = 193.95x + 70.837	0.9999	1.70	5.30
22	Apigenin	320	65.028	6.70-96.00	y = 112.74x + 66.409	0.9998	2.20	6.70

Std: standard; rt: retention time.

Table S5. inter-day variation of polyphenol standards.

Std	Compound	Concentraton µg mL^{-1}	Day 1 ±SD n=2	Day 2 ±SD n=2	Day 3 ±SD n=2	Average of Areas ±SD n=6	RSD%
1	Gallic acid	120	8749.600±2.022E+01	8770.900±8.771E+03	8877.750±1.768E+00	8799.417±6.8670E+01	0.78
2	Protocatechuic acid	50	2299.750±1.520E+01	2336.500±6.081E+00	2383.950±5.586E+00	2340.067±4.2213E+01	1.80
3	(+)-Catechin	120	2540.800±5.233E+00	2566.450±9.192E-01	2584.700±1.980E+00	2563.983±2.2054E+01	0.86
4	Chlorogenic acid	150	10681.000±1.273E+01	10687.000±1.414E+01	10800.500±1.202E+01	10722.833±6.7328E+01	0.63
5	Caffeic acid	30	3792.800±5.233E+00	3915.350±1.690E+01	4068.600±7.071E+00	3925.583±1.3818E+02	3.52
6	(-)-Epicatechin	64	986.350±1.216E+00	994.080±1.004E+00	1001.700±1.556E+00	994.043±7.6751E+00	0.77
7	p-coumaric acid	5	1091.650±1.485E+00	1125.650±4.738E+00	1172.500±5.233E+00	1129.933±4.0595E+01	3.59
8	Ferulic acid	16	1583.900±1.414E-01	1591.300±5.657E-01	1605.300±2.404E+00	1593.500±1.0868E+01	0.68
9	Hyperoside	32	3769.550±2.072E+01	3868.150±1.237E+01	4023.700±1.442E+01	3887.133±1.2813E+02	3.30
10	Isoquercitrin	20	970.415±1.973E+00	974.000±2.404E+00	983.190±3.437E+00	975.868±6.5892E+00	0.68
11	(+)-Rutin Trihydrate	75	1200.200±9.192E+00	1228.400±1.556E+00	1236.450±2.044E+01	1221.683±1.9036E+01	1.56
12	Phloridzin dihydrate	48	2127.250±1.025E+01	2154.050±3.465E+00	2192.750±4.313E+00	2158.017±3.2930E+01	1.53
13	Trans-cinnamic acid	12	1895.150±2.758E+00	1904.300±5.657E-01	1925.250±1.061E+00	1908.233±1.5431E+01	0.81
14	Quercitrin	128	3866.200±3.111E+00	3993.500±1.202E+01	4156.050±1.421E+01	4005.250±1.4528E+02	3.63
15	Myricetin	64	7852.050±3.316E+01	7901.650±4.525E+00	7995.650±9.405E+00	7916.450±7.2935E+01	0.92
16	Daidzein	80	32849.500±4.738E+01	32660.000±2.121E+01	32715.500±5.162E+01	32741.667±9.7422E+01	0.30
17	Naringenin	32	2118.750±4.830E+01	2080.700±2.828E-01	2103.000±1.414E+01	2100.817±1.9119E+01	0.91
18	Genistein	48	7557.650±7.071E-02	7787.400±1.966E+01	8103.000±3.154E+01	7816.017±2.7380E+02	3.50
19	Hesperetin	80	9721.650±1.153E+01	9865.300±4.525E+00	10028.500±1.626E+01	9871.817±1.5353E+02	1.56
20	Naringenin chalcone	48	14759.500±2.051E+01	14712.500±1.626E+01	14685.500±7.071E-01	14719.167±6.8670E+01	0.25
21	Kaempferol	48	18385.000±4.243E+01	18365.500±2.616E+01	18396.500±2.051E+01	18382.333±4.2213E+01	0.09
22	Apigenin	48	12293.000±3.536E+01	12324.000±7.071E+00	12331.500±2.616E+01	12316.167±2.2054E+01	0.17

Std: standard.

Table S6. Intra-day variation of polyphenol standards.

Std	Compound	Concentration µg mL^{-1}	Area 1 ±SD n=2	Area 2 ±SD n=2	Area 3 ±SD n=2	Average of Areas ±SD n=6	RSD%
1	Gallic acid	120	8892.8±4.09E+01	8982.250±1.28E+02	9046.850±8.59E+01	8973.967±7.74E+01	0.86
2	Protocatechuic acid	50	2341.800±3.39E+00	2372.150±2.69E+01	2382.800±2.88E+01	2365.583±2.13E+01	0.90
3	(+)-Catechin	120	2660.450±7.97E+01	2773.100±1.03E+02	2820.950±1.10E+02	2751.500±8.24E+01	2.99
4	Chlorogenic acid	150	10846.000±1.20E+01	10947.500±7.00E+01	11004.500±1.03E+02	10932.667±8.03E+01	0.73
5	Caffeic acid	30	3889.550±5.16E+00	3947.250±2.30E+01	3956.350±2.45E+01	3931.050±3.62E+01	0.92
6	(-)-Epicatechin	64	1033.600±3.82E+00	1075.700±3.97E+01	1093.750±4.31E+01	1067.683±3.09E+01	2.89
7	p-coumaric acid	5	1119.300±1.70E+00	1137.150±6.15E+00	1138.850±7.42E+00	1131.767±1.08E+01	0.96
8	Ferulic acid	16	1657.900±3.11E+00	1726.650±6.46E+01	1754.800±6.99E+01	1713.117±4.98E+01	2.91
9	Hyperoside	32	3854.450±9.97E+00	3900.450±1.34E+01	3900.750±2.24E+01	3885.217±2.66E+01	0.69
10	Isoquercitrin	20	991.735±2.88E+00	998.445±5.87E-01	995.775±4.28E+00	995.318±3.38E+00	0.34
11	(+)-Rutin Trihydrate	75	1214.800±2.01E+01	1215.900±1.56E+01	1233.100±3.41E+01	1221.267±1.03E+01	0.84
12	Phloridzin dihydrate	48	2159.450±1.91E+00	2185.950±2.50E+01	2196.700±2.86E+01	2180.700±1.92E+01	0.88
13	Trans-cinnamic acid	12	1929.350±4.88E+00	1951.500±1.20E+01	1955.800±1.67E+01	1945.550±1.42E+01	0.73
14	Quercitrin	128	3977.650±7.14E+00	4032.650±1.85E+01	4033.200±2.57E+01	4014.500±3.19E+01	0.79
15	Myricetin	64	7676.300±4.67E+00	7651.850±5.95E+01	7709.900±4.98E+01	7679.350±2.91E+01	0.38
16	Daidzein	80	33093.500±2.98E+02	33054.500±5.37E+02	33116.000±6.18E+02	33088.000±3.11E+01	0.09
17	Naringenin	32	2287.850±7.07E-02	2301.250±1.90E+01	2283.350±7.45E+01	2290.817±9.31E+00	0.41
18	Genistein	48	9083.350±1.05E+03	8975.550±1.20E+03	8953.250±1.23E+03	9004.050±6.96E+01	0.77
19	Hesperetin	80	9801.900±2.15E+02	9627.500±1.22E+02	9673.050±2.76E+02	9700.817±9.05E+01	0.93
20	Naringenin chalcone	48	14718.000±3.82E+01	14724.500±7.07E-01	14729.500±6.15E+01	14724.000±5.77E+00	0.04
21	Kaempferol	48	18548.500±2.74E+02	18579.000±3.28E+02	18613.500±3.27E+02	18580.333±3.25E+01	0.18
22	Apigenin	48	12423.000±2.19E+02	12466.000±2.08E+02	12487.500±2.47E+02	12458.833±3.28E+01	0.26

Table S7. %Recovery of HPLC-DAD data.

Standard	Compound	Concentration µg mL^{-1}	% Recovery±SD n= 2	RDS%
1	Gallic Acid	7.031	103.16±5.42E+00	2.20
2	Protocatechuic acid	2.813	91.71±7.73E+00	6.11

3	(+)-Catechin	15.625	92.81±1.20E+01	5.27
4	Chlorogenic acid	2.344	92.47±4.94E+00	5.53
5	Caffeic acid	0.938	91.72±1.21E+01	6.10
6	(-)-Epicatechin	7.031	101.67±1.73E+00	1.17
7	p-coumaric acid	0.469	104.44±1.23E+01	3.07
8	Ferulic acid	1.406	100.36±9.06E-01	0.25
9	Hyperoside	9.375	93.95±2.13E+00	4.41
10	Isoquercitrin	9.375	97.94±5.80E+00	1.47
11	(+)-Rutin Trihydrate	4.688	100.48±1.87E+00	0.34
12	Phloridzin dihydrate	7.031	104.35±7.66E+00	3.01
13	Trans-cinnamic acid	1.875	90.54±2.65E+01	7.02
14	Quercitrin	14.063	95.50±1.23E+01	3.25
15	Myricetin	4.950	101.29±5.95E+00	0.91
16	Daidzein	4.250	99.63±1.81E+01	0.26
17	Naringenin	1.875	99.72±6.42E-01	0.20
18	Genistein	3.645	99.20±2.72E+01	0.57
19	Hesperetin	7.050	99.75±5.46E+00	0.18
20	Naringenin chalcone	4.125	90.78±1.35E+01	6.84
21	Kaempferol	3.000	101.63±5.30E+00	1.15
22	Apigenin	3.300	102.06±6.96E+00	1.44

Figure S5. Overlaid HPLC chromatograms of Pch-Bio-ByP (a1) and Pch-Bio-FinalP (b1) samples registered at λ= 280 nm; λ =320 nm; λ= 370 nm; λ =360 nm; λ =250 nm; the original chromatogram reports the Y-axis in the 0-600 mAU and 0-1200 mAU range, respectively; UV-Vis spectra of detected peaks of Pch-Bio-ByP (a2) and Pch-Bio-FinalP (b2); 2 = protocatechuic acid; 4= chlorogenic acid; 5= caffeic acid; 9= hyperoside; 10= isoquercitrin; 17= naringenin; 24 = chlorogenic acid derivative; 25 = hydroxycinnamic acid derivative; 27 = flavanone derivative.

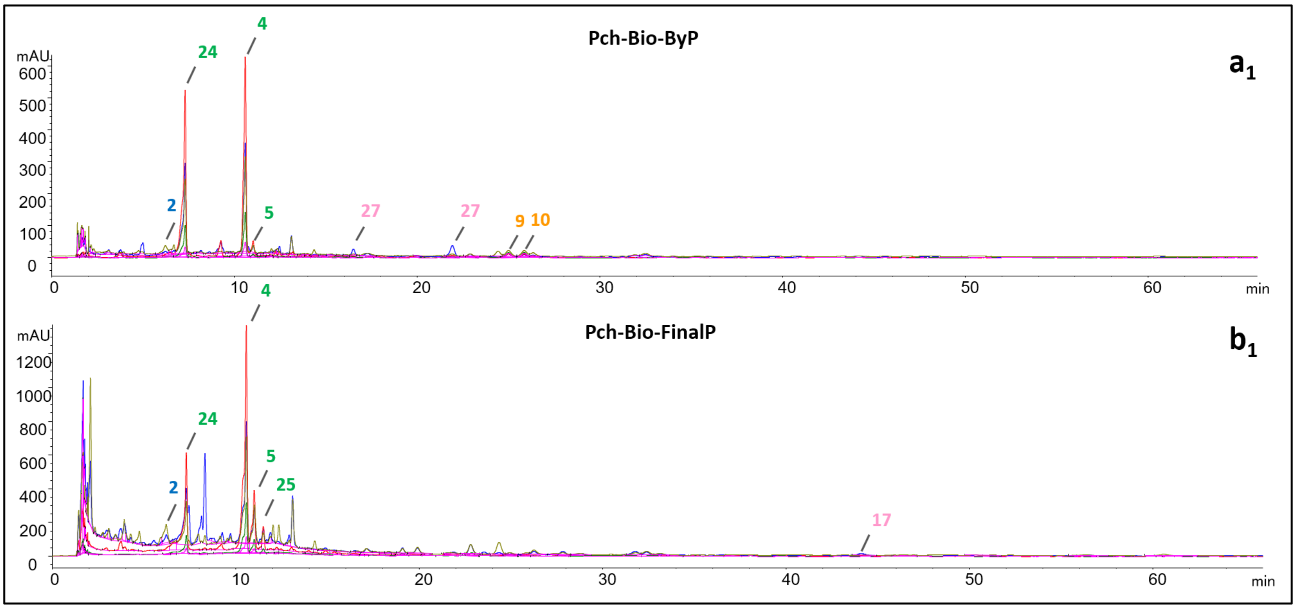

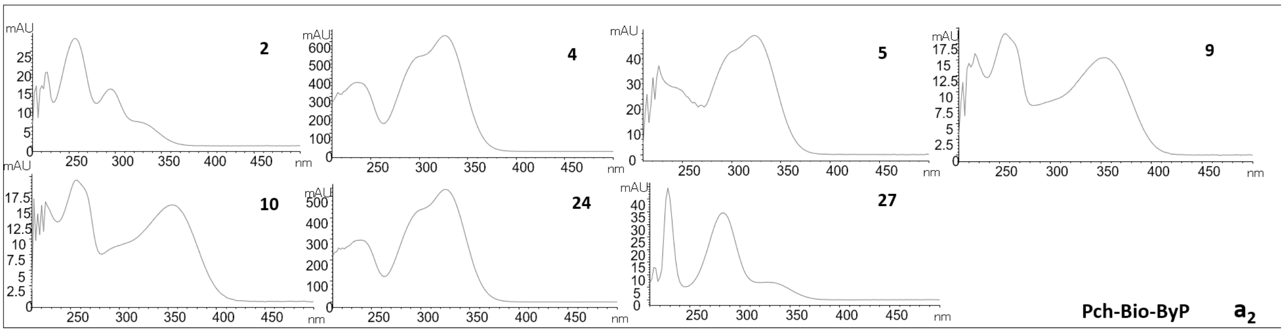

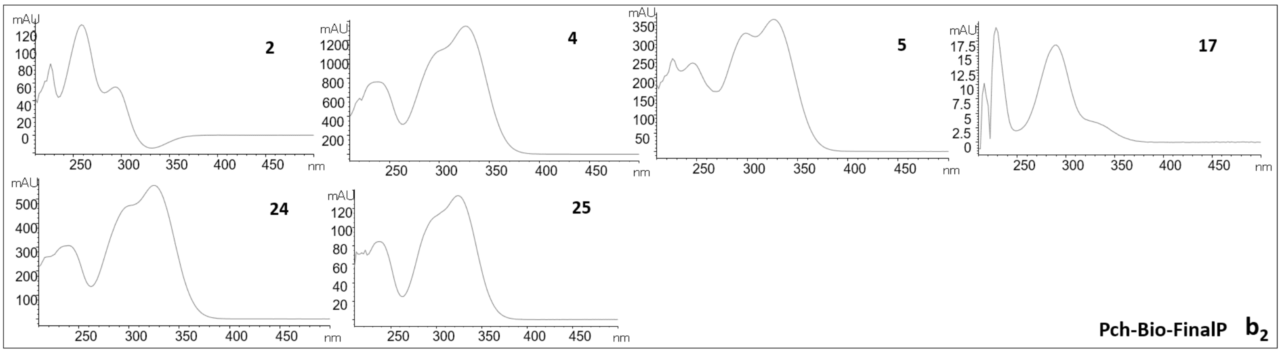

Table S8. Retention times of polyphenols identified in Pch-Bio-ByP and Pch-Bio-FinalP.

Peak	Compound	Reference Standard Retention Time	Pch-Bio-ByP (a1) Retention Time	Pch-Bio-FinalP (b1) retention Time
2	Protocatechuic acid	6.175	6.202	6.201
24	Chlorogenic acid derivative	/	7.302	7.296
4	Chlorogenic acid	10.597	10.593	10.577
5	Caffeic acid	11.522	11.037	11.017
25	Hydroxycinnamic acid derivative	/	/	11.504
27	Flavanone derivative	/	16.509	/

27	Flavanone derivative	/	21.914	/
9	Hyperoside	24.975	24.995	/
10	Isoquercitrin	25.828	25.849	/
17	Naringenin	44.200	/	44.083

Figure S6. Overlaid HPLC chromatograms of Pch-Conv-ByP (a1) and Pch-Conv-FinalP (b1) samples registered at λ= 280 nm; λ =320 nm; λ= 370 nm; λ =360 nm; λ =250 nm; the original chromatogram reports the Y-axis in the 0-600 mAU and 0-1200 mAU range, respectively; UV-Vis spectra of detected peaks of Pch-Conv-ByP (a2) and Pch-Conv-FinalP (b2); 2 = protocatechuic acid; 4= chlorogenic acid; 5= caffeic acid; 9= hyperoside; 10= isoquercitrin; 17= naringenin; 24 = chlorogenic acid derivative; 25 = hydroxycinnamic acid derivative; 27 = flavanone derivative.

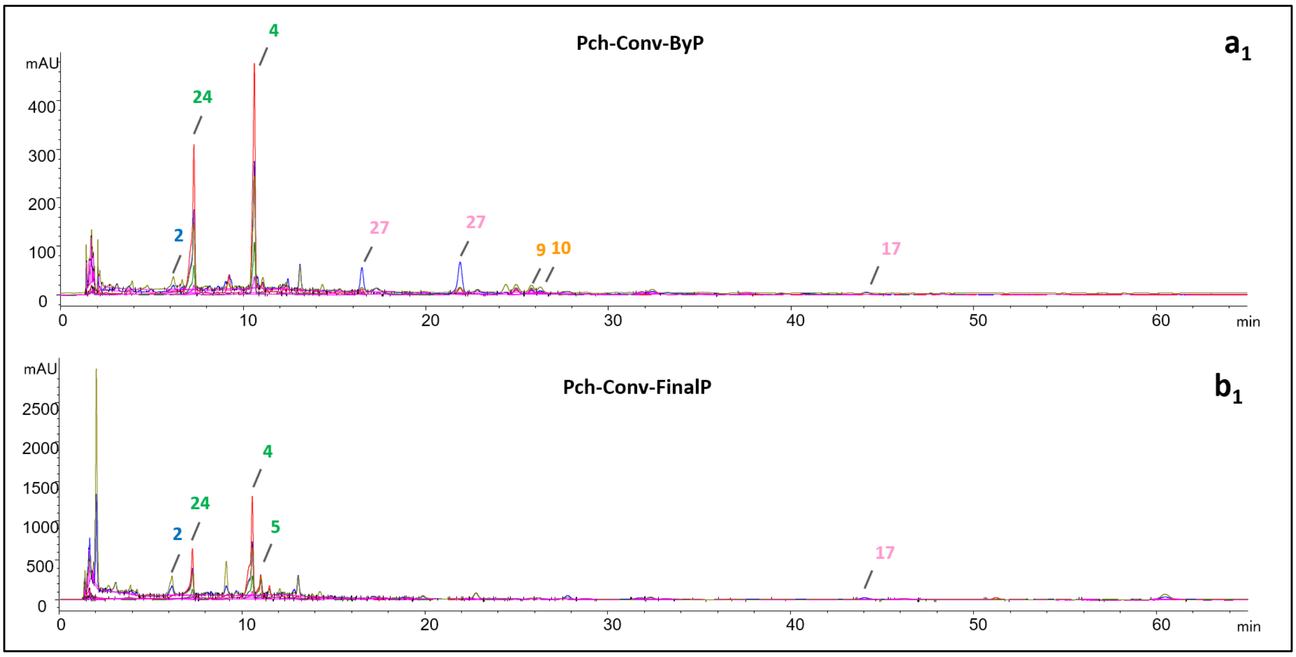

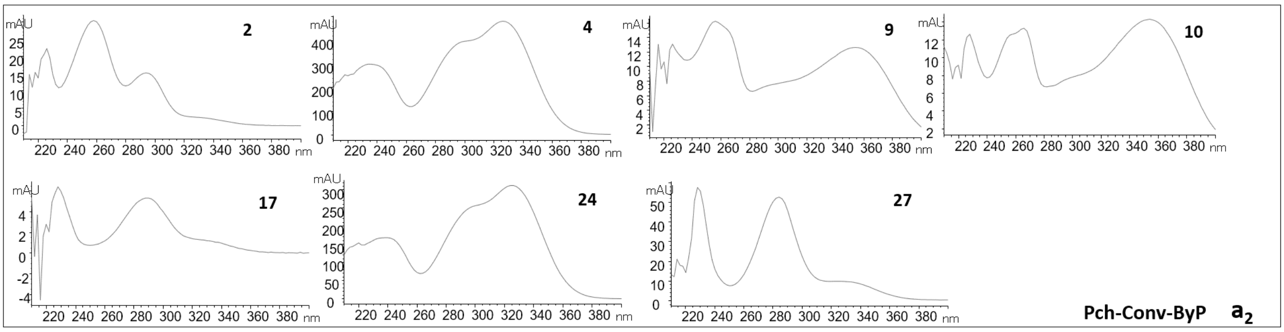

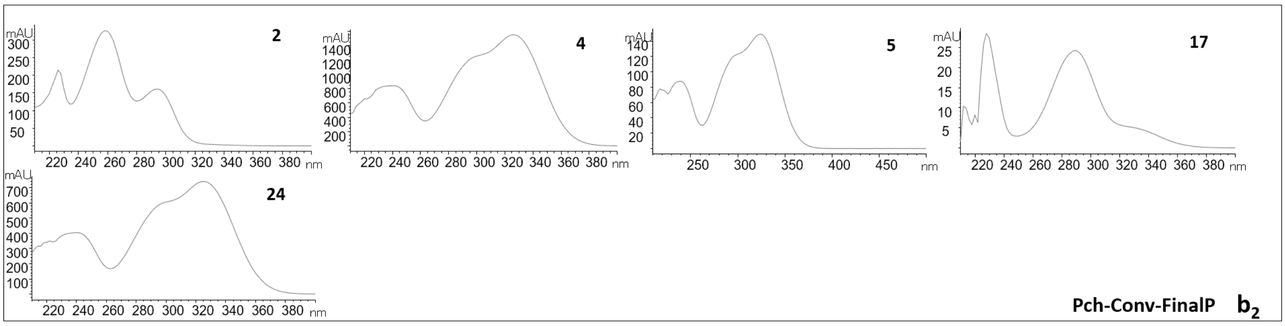

Table S9. Retention times of polyphenols identified in Pch-Conv-ByP and Pch-Conv-FinalP.

Peak	Compound	Reference Standard	Pch-Conv-ByP (a1)	Pch-Conv-FinalP (b1)
		Retention Time	Retention Time	Retention Time
2	Protocatechuic acid	6.175	6.186	6.181
24	Chlorogenic acid derivative	/	7.297	7.295
4	Chlorogenic acid	10.597	10.585	10.575
5	Caffeic acid	11.522	11.027	11.022
27	Flavanone derivative	/	16.494	/
27	Flavanone derivative	/	21.880	/
9	Hyperoside	24.975	24.947	/
10	Isoquercitrin	25.828	25.797	/
17	Naringenin	44.200	44.104	44.032

Figure S7. Overlaid HPLC chromatograms of Ac-Bio-ByP (a1) and Ac-Bio-FinalP (b1) registered at λ= 280 nm; λ =320 nm; λ= 370 nm; λ =360 nm; λ =250 nm; the original chromatogram reports the Y-axis in the 0-600 mAU and 0-1200 mAU range, respectively; UV-Vis spectra of detected peaks of Ac-Bio-ByP (a2) and Ac-Bio-FinalP (b2); 4= chlorogenic acid; 10= isoquercitrin; 11= (+)-rutin trihydrate; 14= quercitrin; 24 = chlorogenic acid derivative; 25 = hydroxycinnamic acid derivative.

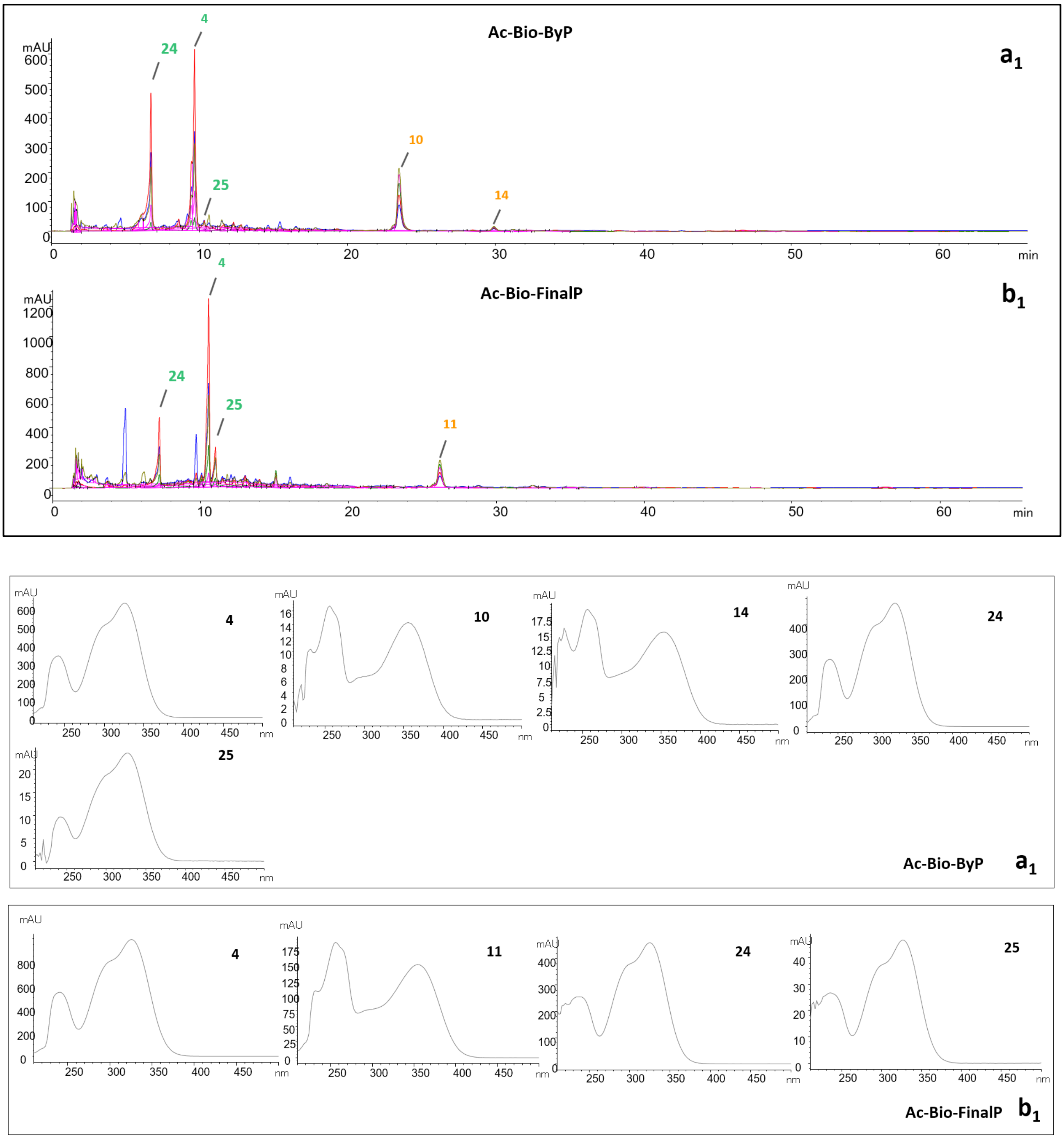

Table S10. Retention times of polyphenols identified in Ac-Bio-ByP and Ac-Bio-ByP.

Peak	Compound	Reference Standard Retention Time	Ac-Bio-ByP (a1) Retention Time	Ac-Bio-FinalP (b1) Retention Time
24	Chlorogenic acid derivative	/	6.798	6.897
4	Chlorogenic acid	10.597	9.703	10.551
25	Hydroxycinnamic acid derivative	/	12.340	13.980
10	Isoquercitrin	25.828	23.784	/
11	(+)-Rutin trihydrate	26.301	/	26.194
14	Quercitrin	32.934	30.269	/

Figure S8. Overlaid HPLC chromatograms of Ac-Conv-ByP (a1) and Ac-Conv-FinalP (b1) registered at λ= 280 nm; λ =320 nm; λ= 370 nm; λ =360 nm; λ =250 nm; the original chromatogram reports the Y-axis in the 0-600 mAU and 0-1200 mAU range, respectively; UV-Vis spectra of detected peaks of Ac-Bio-ByP (a2) and Ac-Bio-FinalP (b2); 4= chlorogenic acid; 10= isoquercitrin; 11= (+)-rutin trihydrate; 14= quercitrin; 24 = chlorogenic acid derivative; 25 = hydroxycinnamic acid derivative.

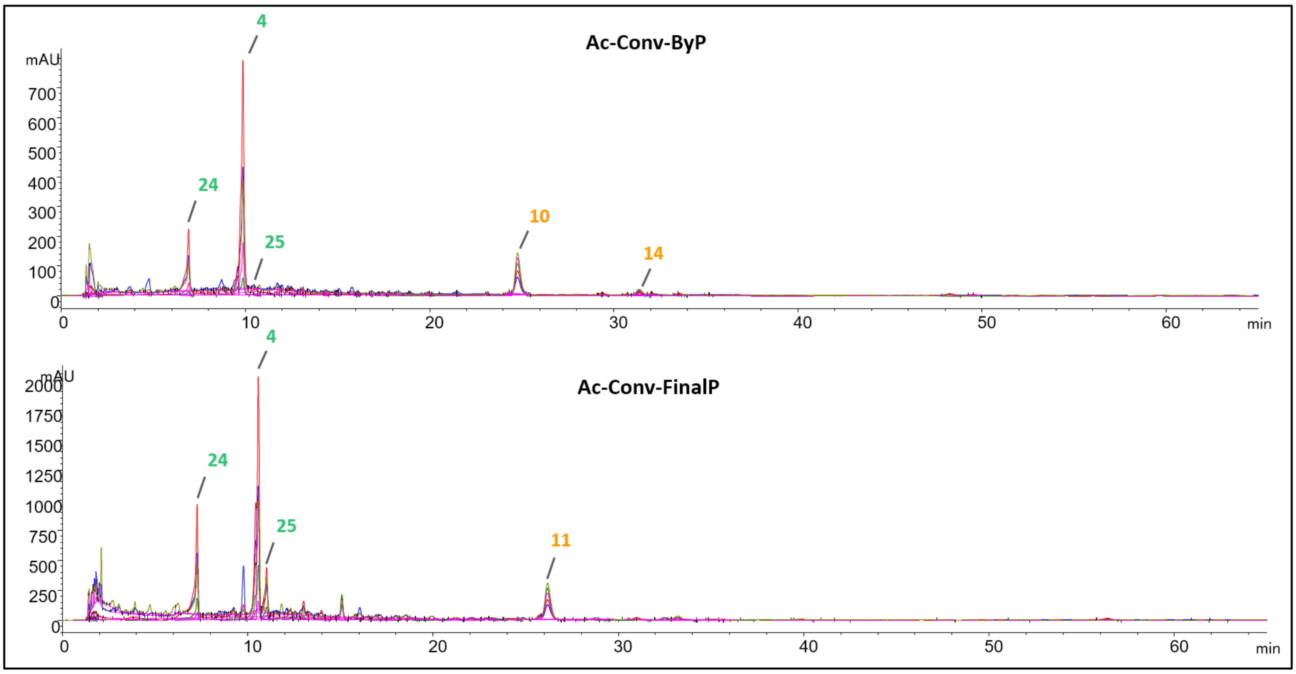

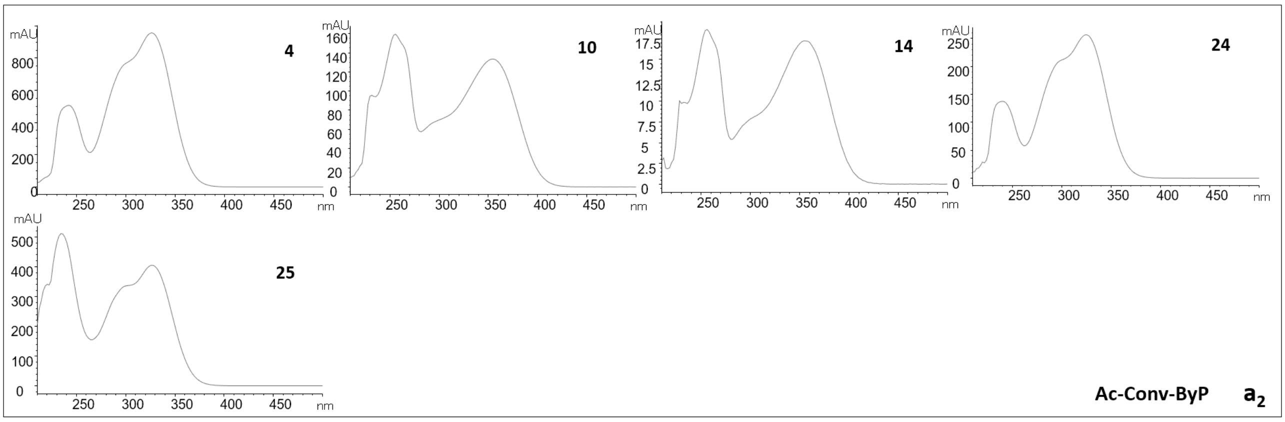

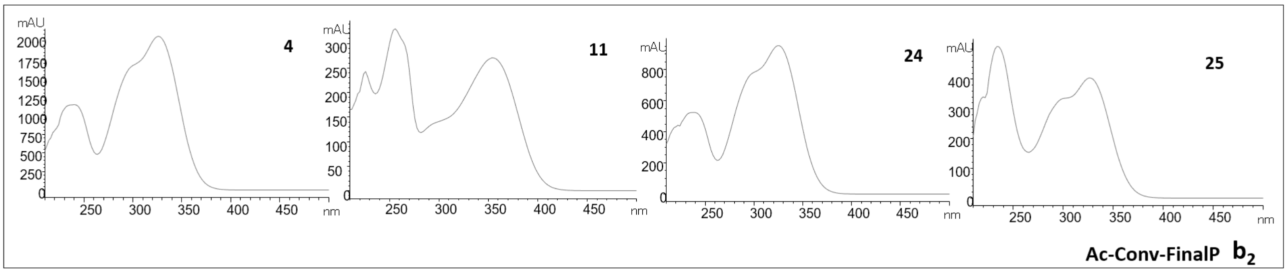

Table S11. Retention times of polyphenols identified in Ac-Conv-ByP and Ac-Conv-ByP.

Peak	Compound	Reference Standard<br>Retention time	Ac-Conv-ByP (a1)<br>Retention Time	Ac-Conv-FinalP (b1)<br>Retention Time
24	Chlorogenic acid derivative	/	6.984	7.298
4	Chlorogenic acid	10.597	9.969	10.580
25	Hydroxycinnamic acid derivative	/	11.934	11.029
10	Isoquercitrin	25.828	23.360	/
11	(+)-Rutin trihydrate	26.301	/	26.285
14	Quercitrin	32.934	30.053	/

Figure S9. Overlaid HPLC chromatograms of T-Bio-ByP (a1) and T-Bio-FinalP (b1) samples registered at λ= 280 nm; λ =320 nm; λ= 370 nm; λ =360 nm; λ =250 nm; the original chromatogram reports the Y-axis in the 0-400 mAU and 0-2000 mAU range, respectively; UV-Vis spectra of detected peaks of T-Bio-ByP (a2) and T-Bio-FinalP (b2); 4= chlorogenic acid; 5= caffeic acid; 11= (+)-rutin trihydrate; 17= naringenin; 25 = hydroxycinnamic acid derivative; 27= flavanone derivative; 28 = flavonol derivative.

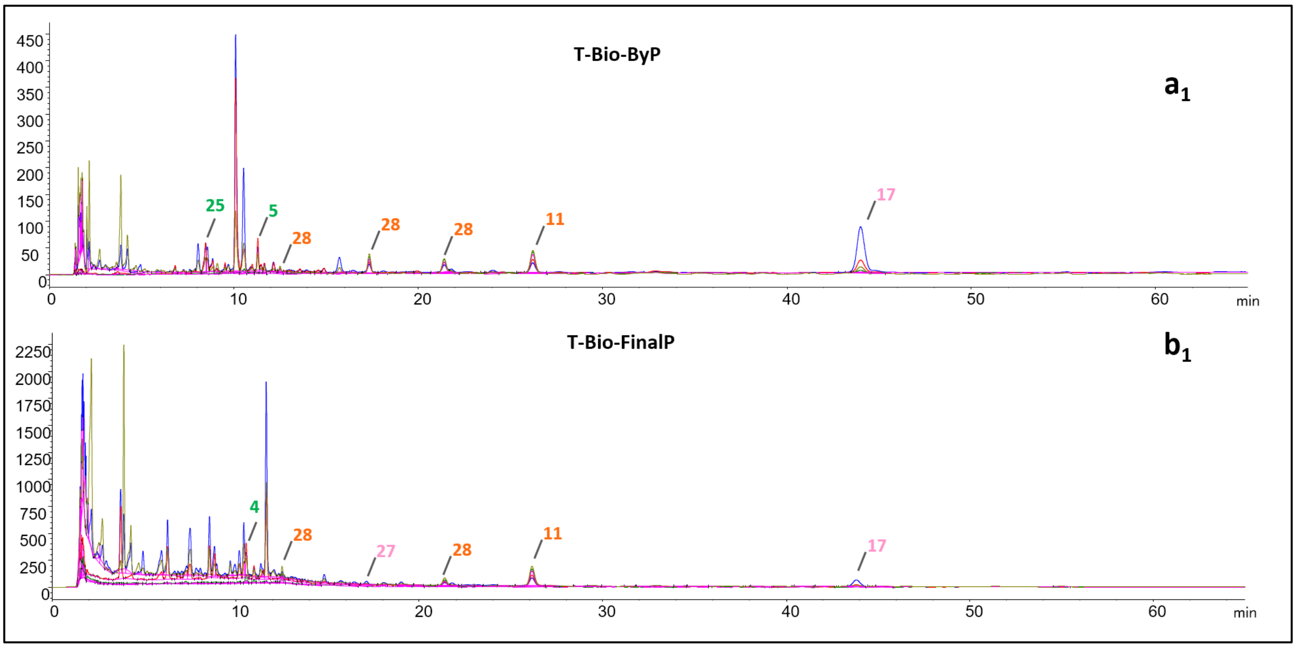
T-Bio-ByP
a1
25
5
28
28
28
11
17
min
T-Bio-FinalP
b1
4
28
27
28
11
17
min

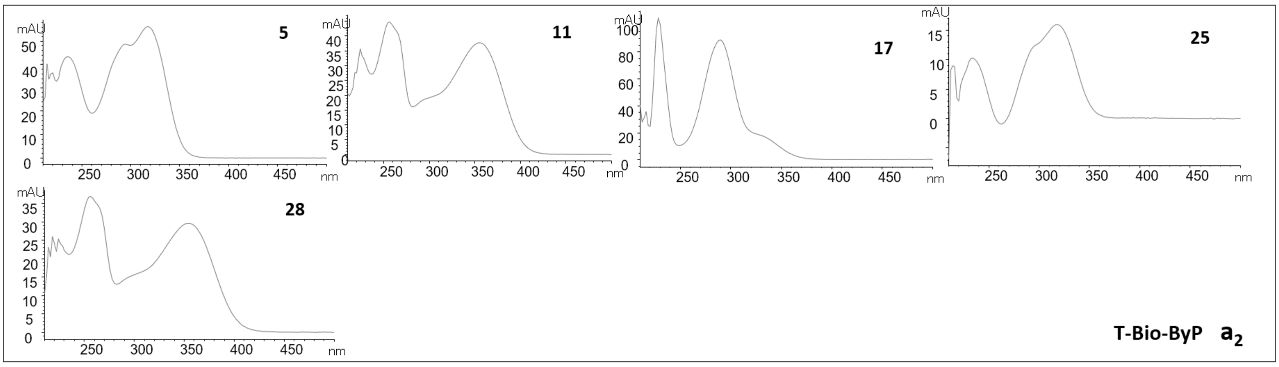
5
11
17
25
28
mAU
nm
T-Bio-ByP a2

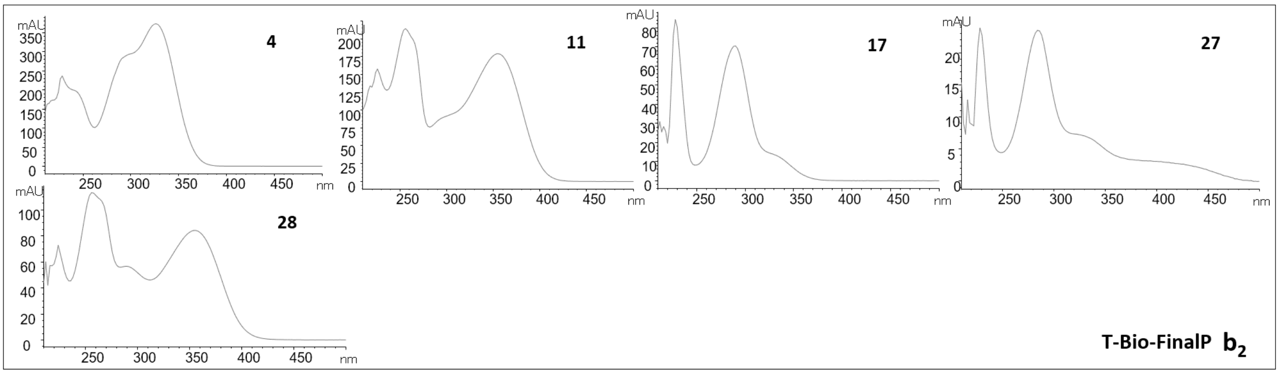
4
11
17
27
28
mAU
nm
T-Bio-FinalP b2

Table S12. Retention times of polyphenols identified in T-Bio-ByP and T-Bio-FinalP.

Peak	Compound	Reference Standard Retention Time	T-Bio-ByP (a1) Retention Time	T-Bio-FinalP (b1) Retention Time
25	Chlorogenic acid derivative	/	9.558	/
4	Chlorogenic acid	10.597	/	10.564
5	Caffeic acid	11.522	11.331	/
28	Flavonol derivative	/	12.527	/
27	Flavanone derivative	/	/	16.448
28	Flavonol derivative	/	17.457	/
28	Flavonol derivative	/	21.602	21.575
11	(+)-Rutin trihydrate	26.301	26.270	26.214
17	Naringenin	44.200	43.709	43.673

Figure S10. Overlaid HPLC chromatograms of T-LI-ByP (a1) and T-Conv-FinalP (b1) samples registered at λ= 280 nm; λ =320 nm; λ= 370 nm; λ =360 nm; λ =250 nm; the original chromatogram reports the Y-axis in the 0-400 mAU and 0-2000 mAU range, respectively; UV-Vis spectra of detected peaks of T-LI-ByP (a2) and T-Conv-FinalP (b2); 4= chlorogenic acid; 5= caffeic acid; 11= (+)-rutin trihydrate; 17= naringenin; 25 = hydroxycinnamic acid derivative; 27= flavanone derivative; 28 = flavonol derivative.

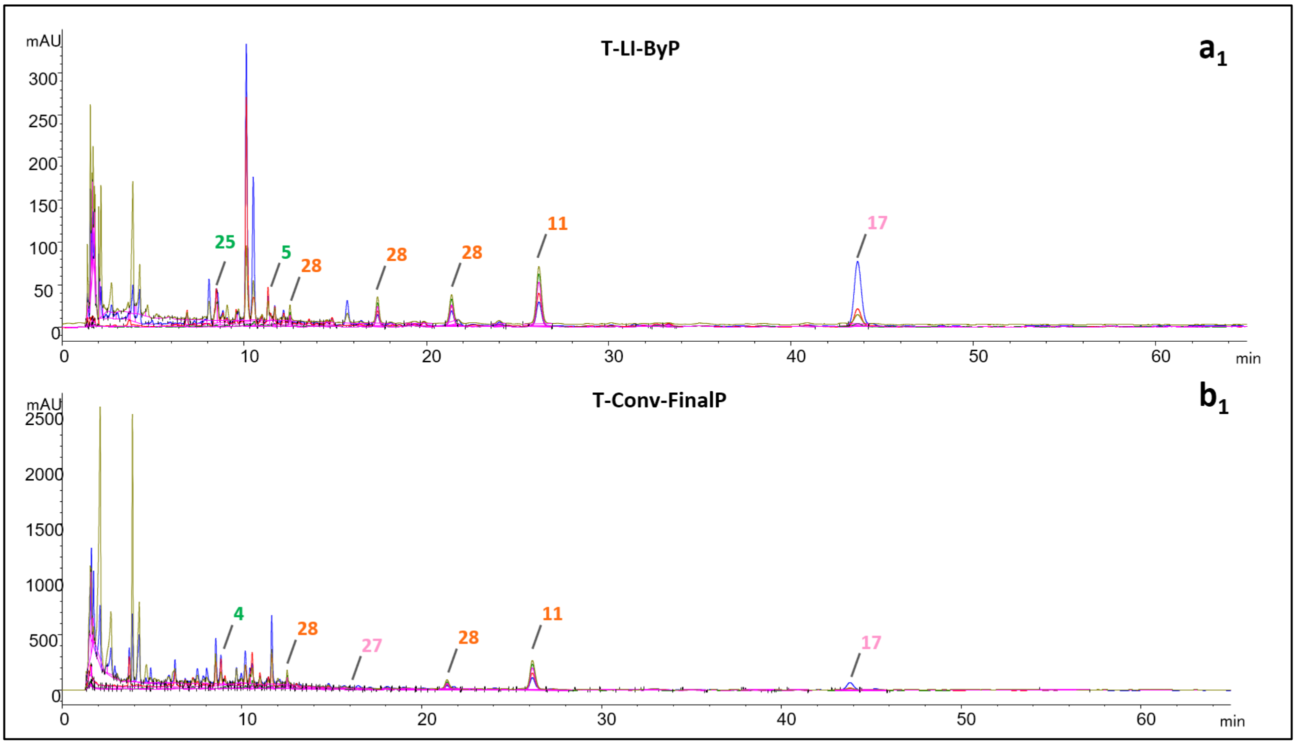

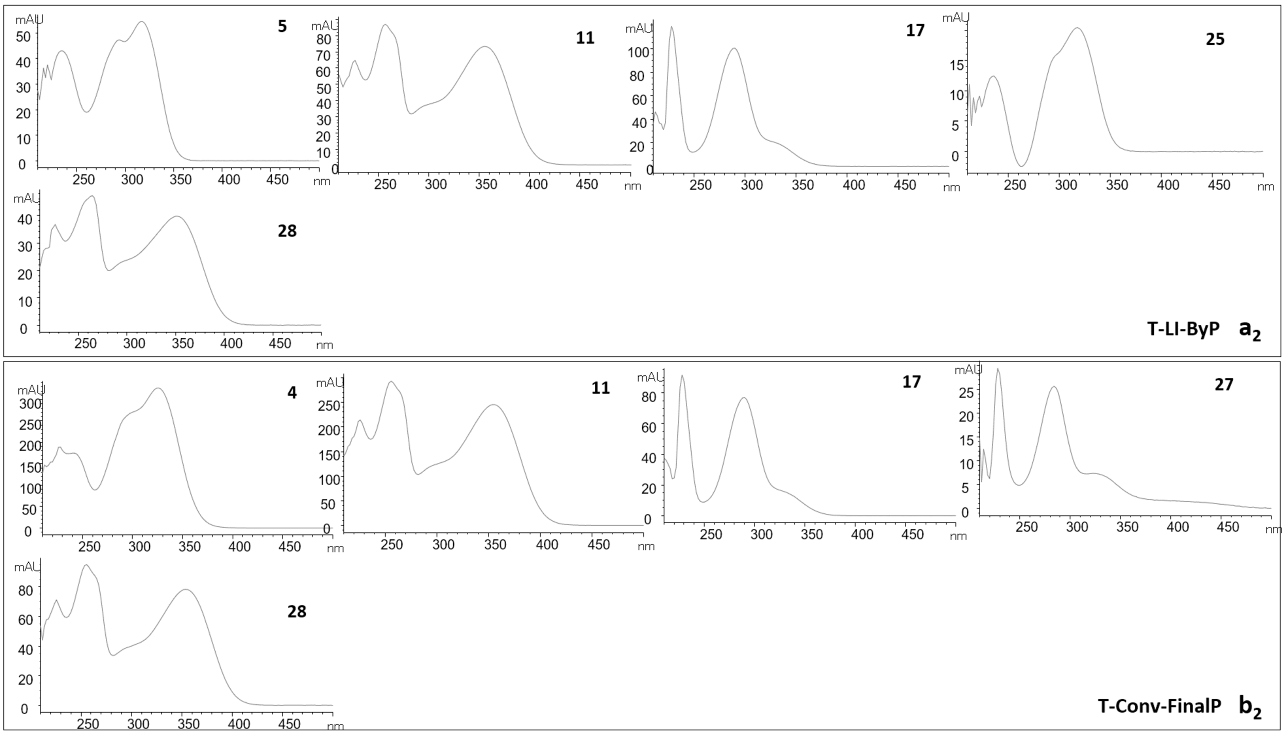

Table S13. Retention times of polyphenols identified in T-LI-ByP and T-Conv-FinalP.

Peak	Compound	Reference Standard Retention Time	T-LI-ByP (a1) Retention Time	T-Conv-FinalP (b1) Retention Time
25	Chlorogenic acid derivative	/	9.552	/
4	Chlorogenic acid	10.597	/	10.556

5	Caffeic acid	11.522	11.317	/
28	Flavonol derivative	/	12.523	/
27	Flavanone derivative	/	/	16.444
28	Flavonol derivative	/	17.349	/
28	Flavonol derivative	/	21.431	21.277
11	(+)-Rutin trihydrate	26.301	26.219	26.073
17	Naringenin	44.200	43.861	43.707

Figure S11. Overlaid HPLC chromatograms of Apl-Bio-ByP (a1), Apl-Conv-ByP (b1) and Apl-FinalP (c1) samples registered at λ= 280 nm; λ =320 nm; λ= 370 nm; λ =360 nm; λ =250 nm; the original chromatogram reports the Y-axis in the 0-250 mAU and 0-1000 mAU range, respectively; UV-Vis spectra of detected peaks of Apl-Bio-ByP (a2), Apl-Conv-ByP (b2), and Apl-FinalP (c2); 2= protocatechuic acid; 4 = chlorogenic acid; 5 = caffeic acid; 6= (-)-epicatechin; 9= hyperoside; 10= isoquercitrin; 11= (+)-rutin trihydrate; 12= phloridzin dihydrate; 14= quercitrin; 15= myricetin; 25 = hydroxycinnamic acid derivatives; 29 = dihydrochalcone (phloridzin) derivative.

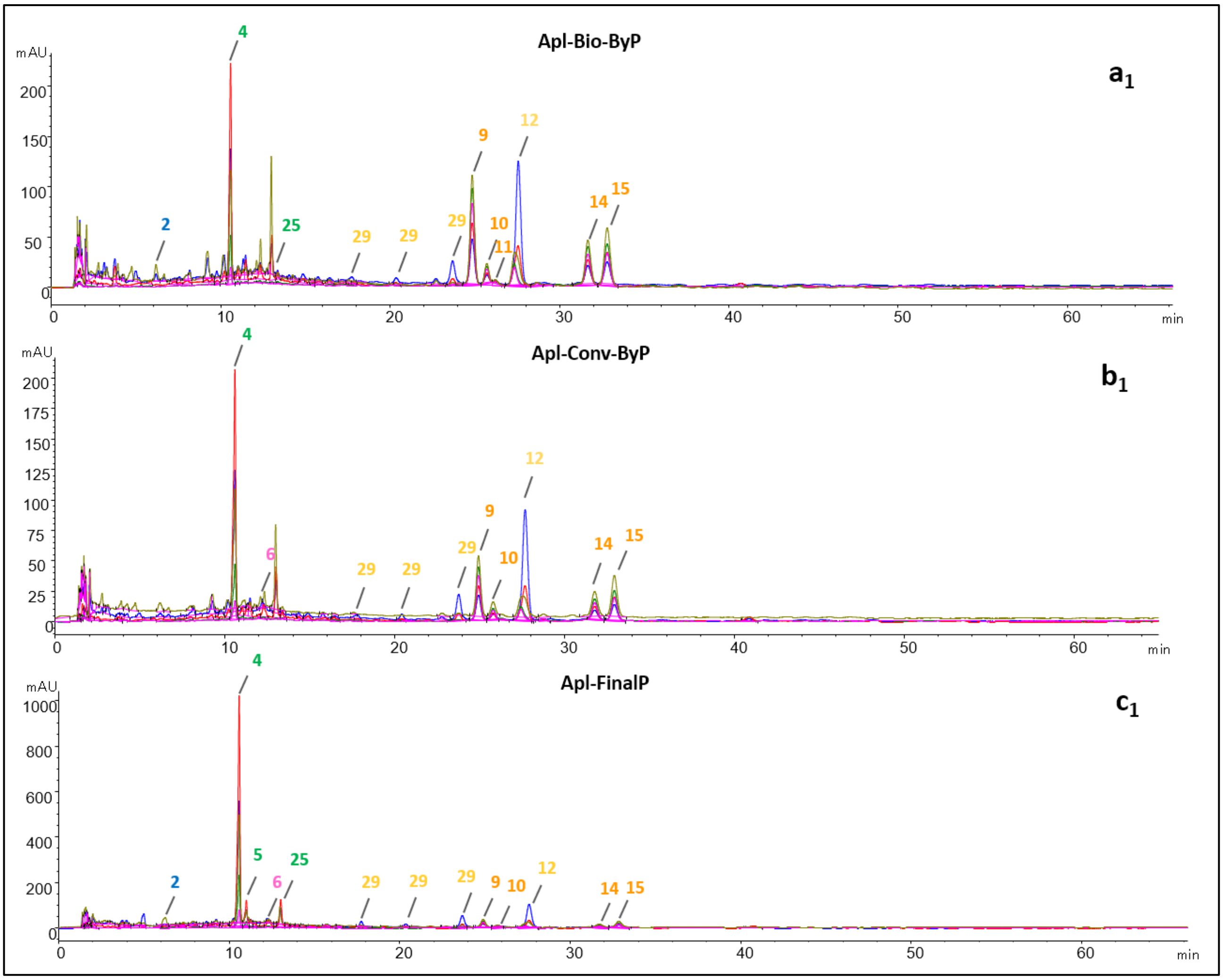
Apl-Bio-ByP
a1
mAU
min
4
2
25
29
9
12
10
11
14
15
Apl-Conv-ByP
b1
6
Apl-FinalP
c1
5

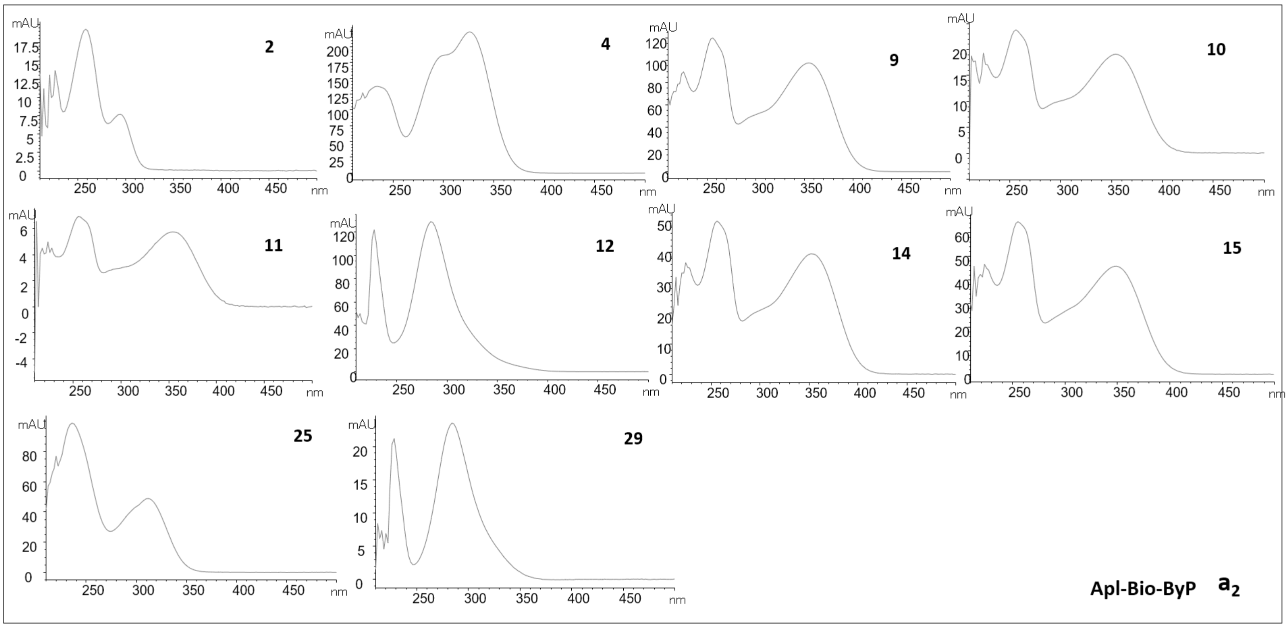
mAU
nm
2
4
9
10
11
12
14
15
25
29
Apl-Bio-ByP
a2

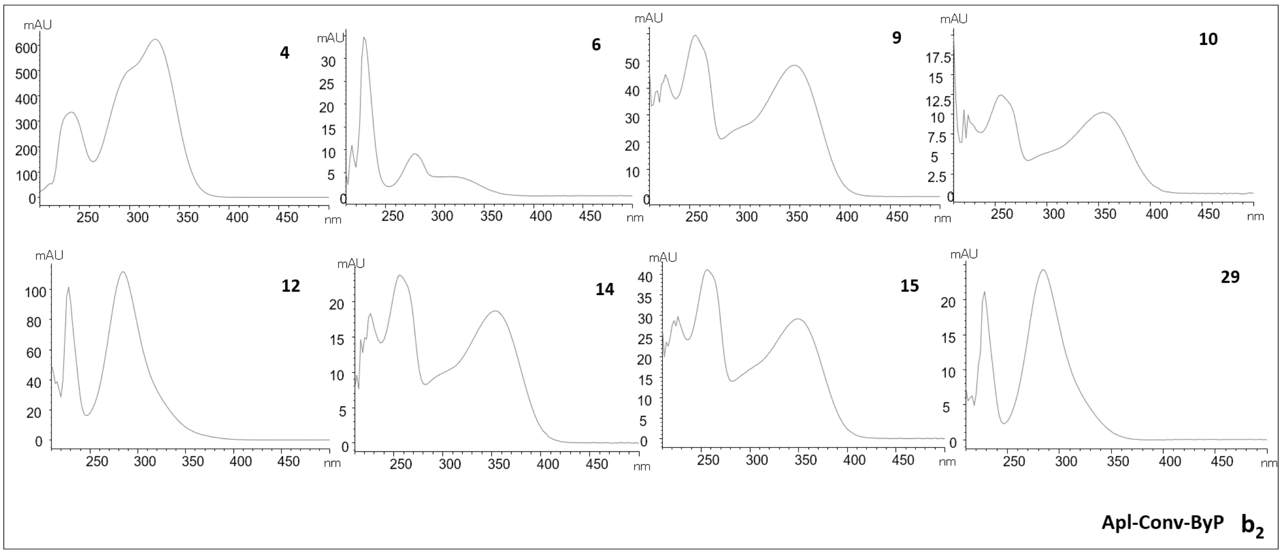

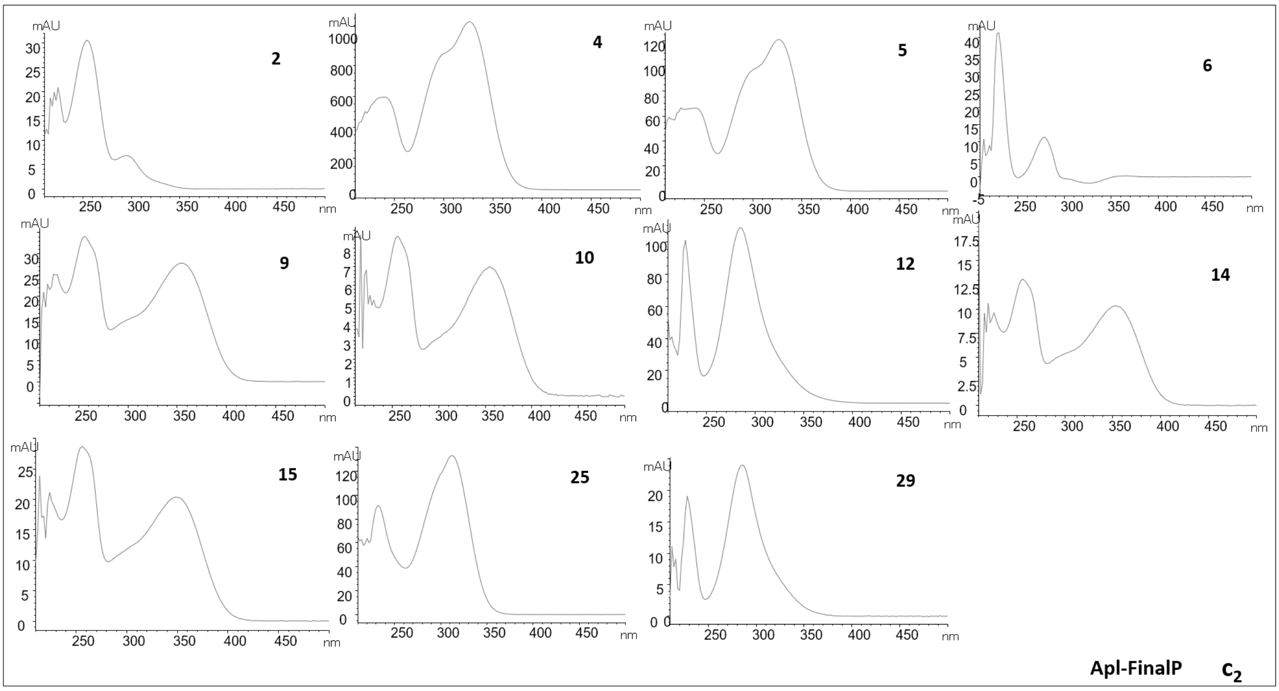

Table S14. Retention times of polyphenols identified in Apl-Bio-ByP, Apl-Conv-ByP, and Apl-FinalP.

Peak	Compound	Reference Standard Retention Time	Apl-Bio-ByP (a1) Retention Time	Apl-Conv-ByP (b1) Retention Time	Apl-Bio-FinalP (c1) Retention Time
2	Protocatechuic acid	6.175	6.178	/	6.182
4	Chlorogenic acid	10.597	10.574	10.585	10.574
5	Caffeic acid	11.522	/	/	11.013
6	(-)-Epicatechin	12.204	/	12.204	12.199

25	Hydroxycinnamic acid derivatives	/	13.367	/	13.380
29	Phlorizin dihydrate derivative	/	17.738	17.792	17.741
29	Phlorizin dihydrate derivative	/	20.361	20.424	20.365
29	Phlorizin dihydrate derivative	/	23.677	23.765	23.674
9	Hyperoside	24.975	24.853	24.963	24.940
10	Isoquercitrin	25.828	25.726	25.826	25.807
11	(+)-Rutin trihydrate	26.301	26.203	/	/
12	Phloridzin dihydrate	27.814	27.577	27.692	27.679
14	Quercitrin	32.934	31.665	31.801	31.776
15	Myricetin	33.915	32.818	32.956	32.930

Figure S12. Quantitative phenolic characterization of peach samples.

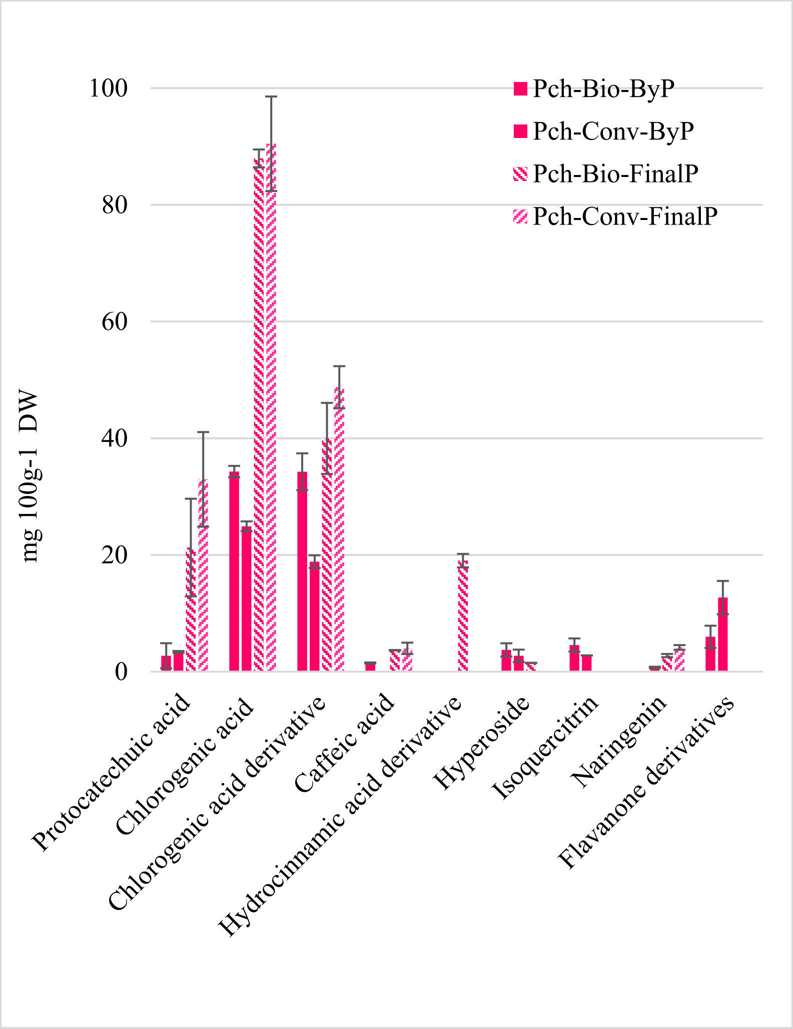

Table S15. Quantification of polyphenols in each peach sample expressed in mg 100g^{-1} DW.

Compound	Pch-Bio-ByP<br>mg 100g^{-1} DW±SD<br>n=3	Pch-Conv- ByP<br>mg 100g^{-1} DW±SD<br>n=3	Pch-Bio-FinalP<br>mg 100g^{-1} DW±SD<br>n=3	Pch-Conv- FinalP<br>mg 100g^{-1} DW±SD<br>n=3
Protocatechuic acid	2.73±2.15E+00	3.40±1.45E-01	21.27±8.37E+00	32.95±8.11E+00
Chlorogenic acid	34.29±9.71E-01	24.92±8.37E-01	87.96±1.53E+00	90.47±8.10E+00
Chlorogenic acid derivative	34.26±3.15E+00	18.85±1.09E+00	39.98±6.09E+00	48.75±3.61E+00
Caffeic acid	1.50±8.38E-02	/	3.67±2.93E-02	4.00±9.74E-01
Hydroxycinnamic acid derivative	/	/	19.02±1.16E+00	/
Hyperoside	3.73±1.13E+00	2.72±1.05E+00	1.50±2.65E-02	/
Isoquercitrin	4.57±1.13E+00	2.78±1.49E-02	/	/
Naringenin	/	0.74±1.10E-01	2.76±2.78E-01	4.15±4.02E-01
Flavanone derivatives	5.98±1.90E+00	12.70±2.85E+00	/	/

Figure S13. Quantitative phenolic characterization of apple samples.

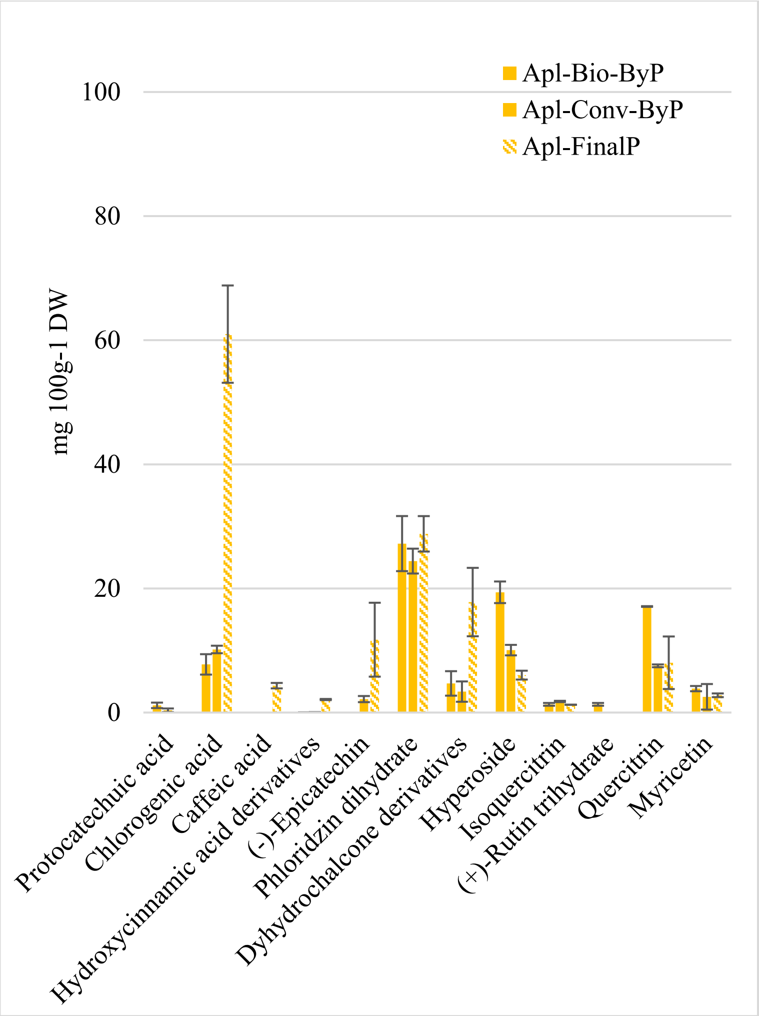

Table S16. Quantification of polyphenols in each apple sample expressed in mg 100g^{-1} DW.

Compound	Apl-Bio- ByP mg 100g^{-1} DW±SD n=3	Apl-Conv-SP mg 100g^{-1} DW±SD n=3	Apl- FinalP mg 100g^{-1} DW±SD n=3
Protocatechuic acid	1.16±4.41E-01	0.37±2.90E-01	/
Chlorogenic acid	7.76±1.65E+00	10.17±6.07E-01	60.98±7.84E+00
Caffeic acid	/	/	4.33±4.40E-01

Hydroxycinnamic acid derivatives	0.03±8.00E-03	0.04±2.58E-02	2.10±9.39E-02
(-)-Epicatechin	/	2.17±4.93E-01	11.74±5.96E+00
Phloridzin dihydrate	27.23±4.44E+00	24.41±2.00E+00	28.80±2.86E+00
Dihydrochalcone derivatives	4.69±1.97E+00	3.38±1.64E+00	17.80±5.51E+00
Hyperoside	19.38±1.74E+00	10.06±8.45E-01	6.03±7.14E-01
Isoquercitrin	0.68±1.68E-01	1.79±1.07E-01	1.44±2.52E-01
(+)-Rutin trihydrate	1.32±2.23E-01	/	/
Quercitrin	17.09±5.77E-02	7.53±2.25E-01	8.02±4.23E+00
Myricetin	3.85±4.22E-01	2.54±2.06E+00	2.78±2.99E-01

Figure S14. Quantitative phenolic characterization of apricot samples.

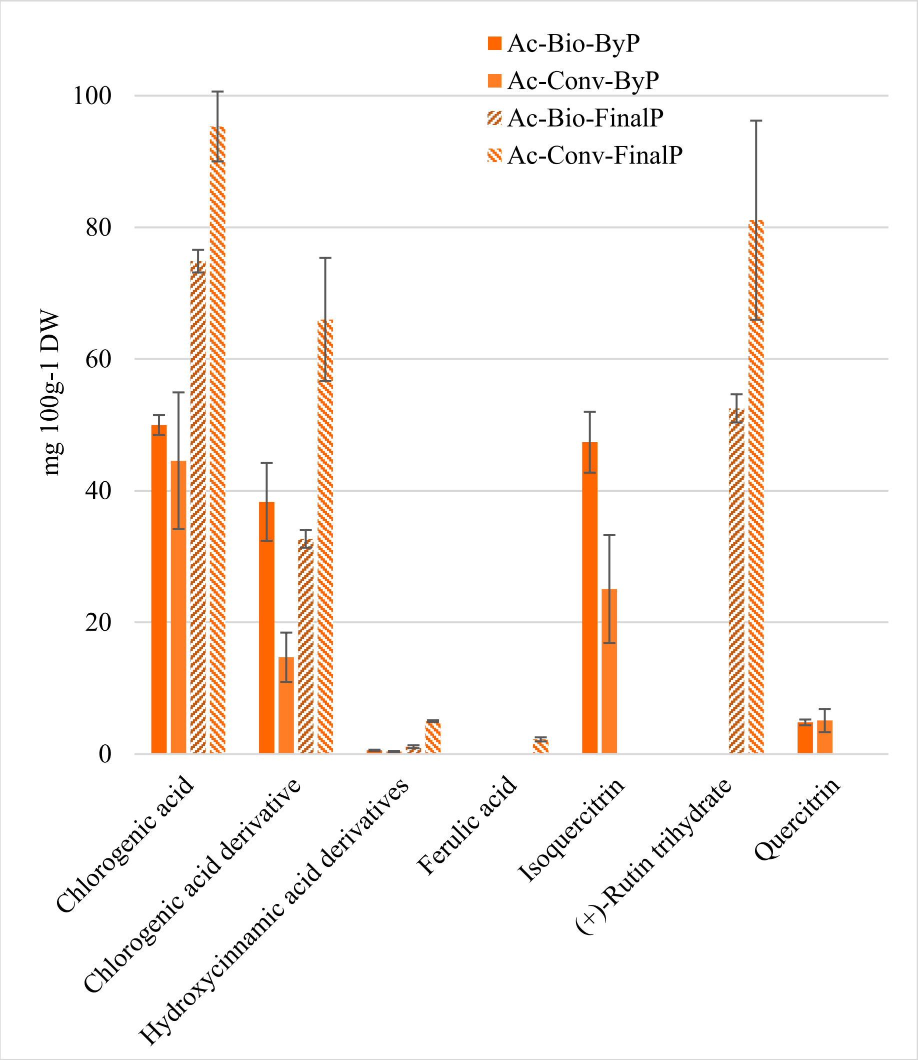

Table S17. Quantification of polyphenols in each apricot sample expressed in mg 100g^{-1} DW.

	**Ac-Bio- ByP**	**Ac-Conv- ByP**	**Ac-Bio- FinalP**	**Ac-Conv- FinalP**
**Compound**	**mg 100g^{-1} DW±SD n=3**	**mg 100g^{-1} DW±SD n=3**	**mg 100g^{-1} DW±SD n=3**	**mg 100g^{-1} DW±SD n=3**
Chlorogenic acid	49.95±1.51E+00	44.55±1.04E+01	74.86±1.72E+00	95.32±5.31E+00
Chlorogenic acid derivative	38.30±5.92E+00	14.70±3.75E+00	32.67±1.32E+00	66.00±9.35E+00
Hydroxycinnamic acid derivatives	0.57±9.12E-02	0.38±1.01E-01	1.11±2.19E-01	5.00±1.34E-01
Ferulic acid	/	/	/	2.22±3.11E-01
Isoquercitrin	47.37±4.63E+00	25.07±8.20E+00	/	/
(+)-Rutin trihydrate	/	/	52.50±2.14E+00	81.09±1.51E+01

Quercitrin	4.80±4.39E-01	5.09±1.76E+00	/	/

Figure S15. Quantitative phenolic characterization of tomato samples.

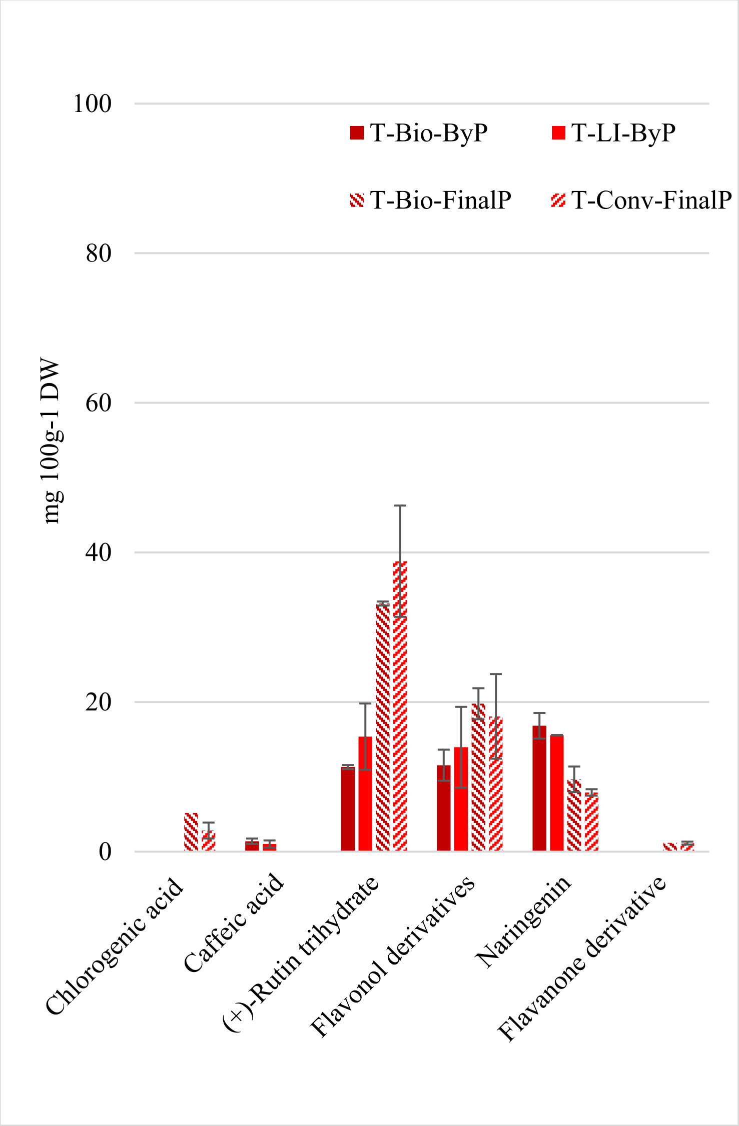

Table S18. Quantification of polyphenols in each tomato sample expressed in mg $100g^{-1}$ DW.

Compound	T-Bio- ByP<br>mg 100g-1 DW±SD<br>n=3	T-LI- ByP<br>mg 100g-1 DW±SD<br>n=3	T-Bio- FinalP<br>mg 100g-1 DW±SD<br>n=3	T-Conv- FinalP<br>mg 100g-1 DW±SD<br>n=3
Chlorogenic acid	/	/	5.18±2.22E+00	2.82±1.06E+00
Caffeic acid	1.39±3.62E-01	1.02±4.82E-01	/	/
(+)-Rutin Trihydrate	11.31±2.71E-01	15.37±4.44E+00	33.17±8.61E+00	38.83±7.44E+00
Flavonol derivatives	11.56±2.07E+00	13.96±5.39E+00	19.78±5.82E+00	18.08±5.66E+00
Naringenin	16.83±1.71E+00	15.57±2.41E-02	9.67±1.22E+00	7.92±4.37E-01
Flavanaone derivative	/	/	1.18±3.57E-01	1.14±1.98E-01

Figure S16. Graphs showing the correlations between the sum of the individual quantities of phenolic compounds (SPC), determined by HPLC, and the total phenolic content (TPC) values. Each graph refers to the results obtained for both fruit by-products and the final products of each sample type (A) peach samples; B) apricot samples; C) apple samples; D) tomato samples). Results are expressed as the mean value of three replicates (n=3).

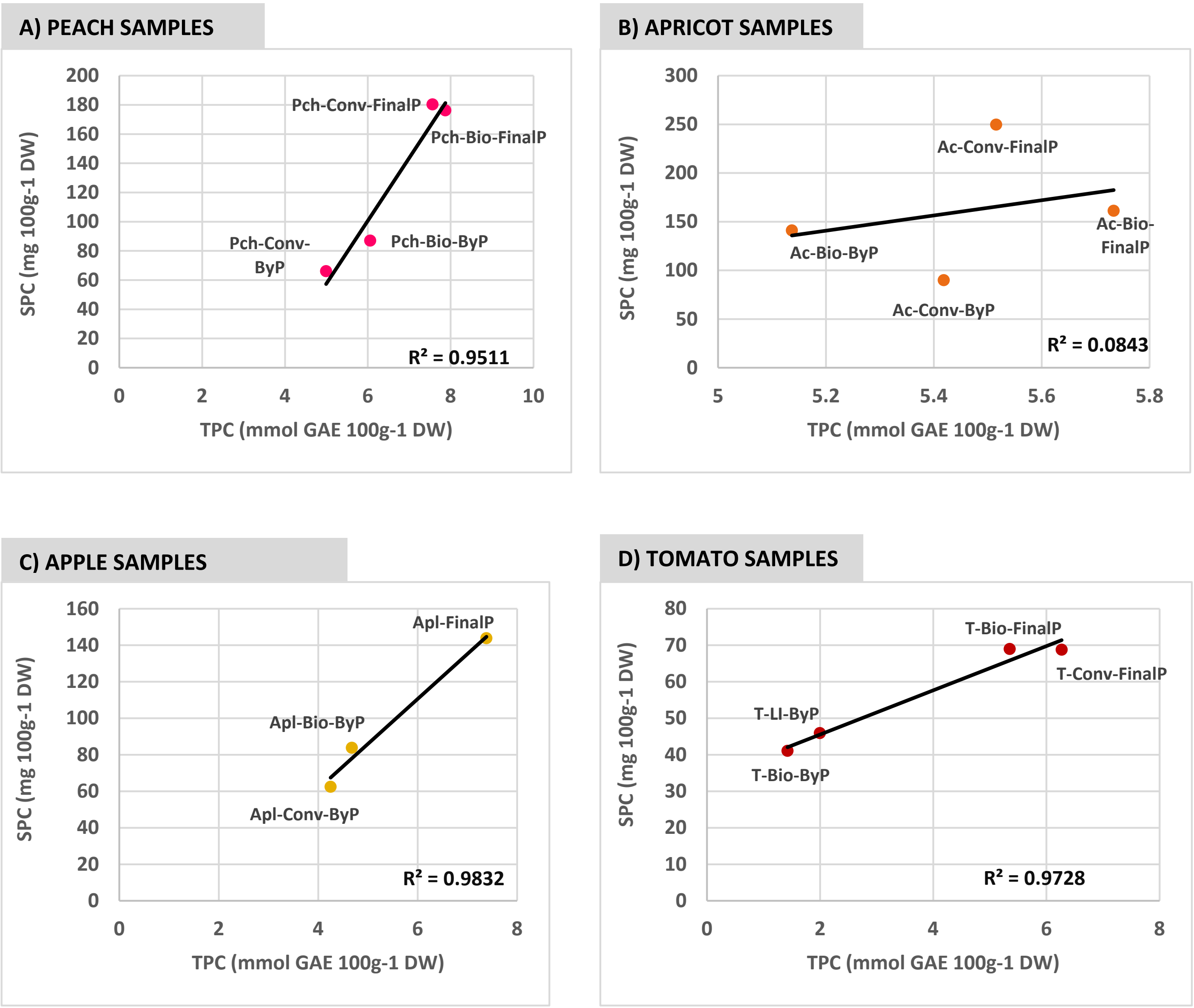

Figure S17. Characteristic UV-Vis spectra of standards classified according to their phenolic classes/families.

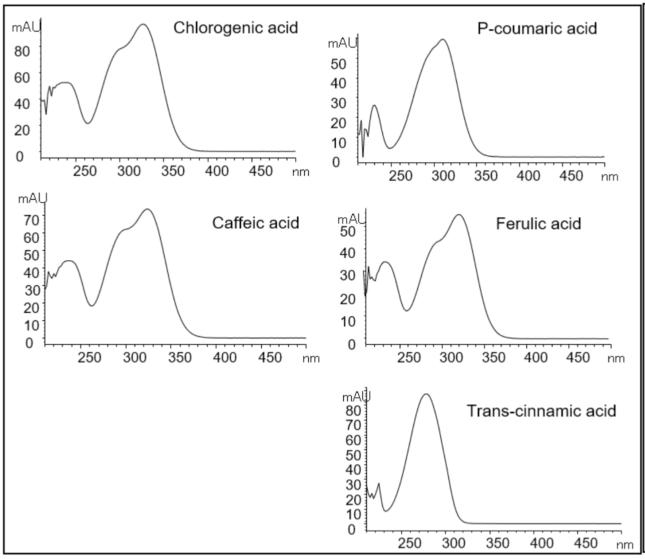

Hydroxycinnamic acid

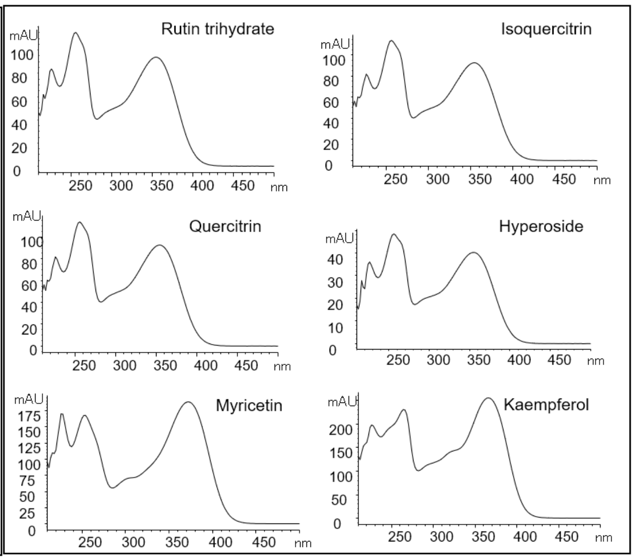

Falavonols

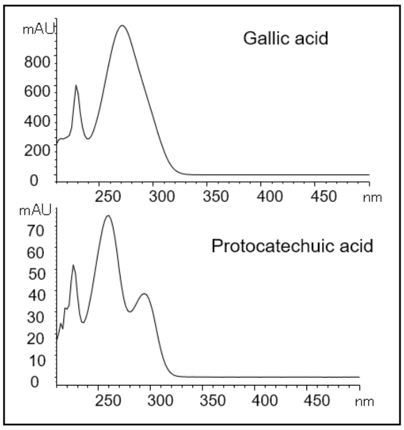

Hydrobenzoic acid

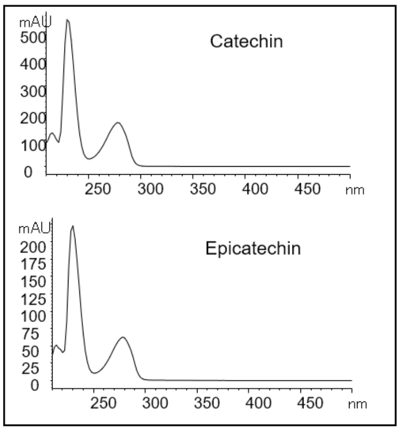

Flavan-3-ols

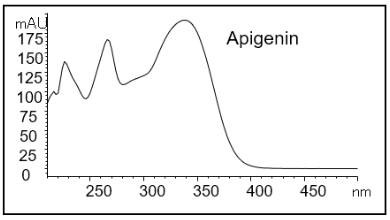

Flavones

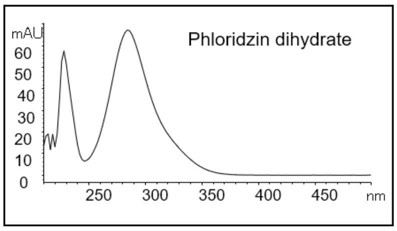

Dihiyrochalcones

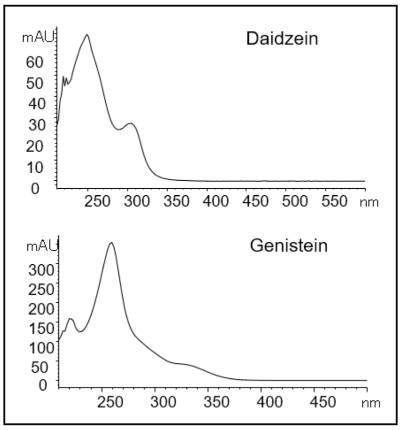

Isoflavones

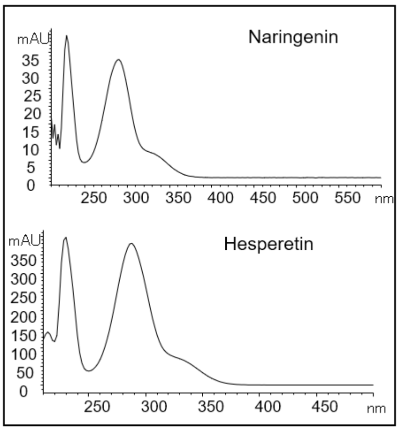

Flavanones

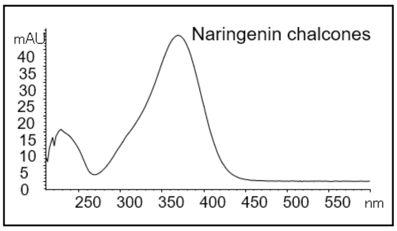

Chalcones
